# Revealing the Mechanism of Exciton Spontaneous Separation at Room Temperature for Efficient Photocatalytic Hydrogen Peroxide Synthesis

**DOI:** 10.1002/advs.202503929

**Published:** 2025-05-19

**Authors:** Pan Jiang, Yuyan Huang, Xiangqiong Jiang, Huijie Yan, Shufang Liu, Zuoming Chen, Xin Wu, Xiantai Zhou, Yu‐Xin Ye, Gangfeng Ouyang

**Affiliations:** ^1^ School of Chemical Engineering and Technology, ICGME Sun Yat‐sen University Zhuhai 519082 P. R. China; ^2^ Key Laboratory of Bioinorganic and Synthetic Chemistry of Ministry of Education LIFM, School of Chemistry IGCME Sun Yat‐sen University Guangzhou 510275 P. R. China; ^3^ Southern Marine Science and Engineering Guangdong Laboratory Zhuhai Guangdong 519082 P. R. China

**Keywords:** charge transfer, donor‐acceptor conjugated polymers, exciton dissociation, H_2_O_2_ synthesis, photocatalysis

## Abstract

The photocatalytic synthesis of hydrogen peroxide (H_2_O_2_) at room temperature has garnered significant attention as an environmentally friendly alternative to traditional anthraquinone oxidation processes. However, the low exciton dissociation efficiency at room temperature often hinders photocatalytic performance. In this study, it is demonstrated that tuning the substitution sites of electron donors in Donor‐Acceptor (D‐A) conjugated polymers can significantly enhance exciton dissociation by reducing exciton activation energy, which facilitates the spontaneous separation of excitons at room temperature. For comparison, materials with exciton separation energies ≈89 meV exhibit a hydrogen peroxide production rate of 2692 µmol·g^−1^·h^−1^. In contrast, the main material developed in this work, O‐PTAQ, demonstrates a substantially lower exciton separation energy of 22 meV, resulting in a hydrogen peroxide production rate of 4989 µmol·g^−1^·h^−1^ under ambient conditions, outperforming most reported organic semiconductors. This enhancement is attributed to the increased electron delocalization in the electron donors, which lowers exciton activation energy to promote efficient exciton separation. The findings highlight the critical role of molecular‐level structural tuning in enhancing exciton dissociation, providing a promising strategy for the development of high‐efficiency photocatalysts for sustainable H_2_O_2_ production.

## Graphical abstract

This study identifies that replacing electron‐donating units at different positions enhances the delocalization of the electron cloud in the electron donors, which in turn increases the delocalization of the overall material. The exciton activation energy is effectively reduced, promoting the spontaneous dissociation of excitons at room temperature and enabling highly efficient photocatalytic synthesis of H_2_O_2_.

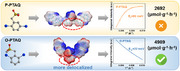

## 1 Introduction

Hydrogen peroxide (H_2_O_2_) is a crucial chemical with diverse applications, including medical sterilization, wastewater treatment, and energy storage.^[^ ^1^ ^]^ However, traditional industrial methods for H_2_O_2_ production, such as the anthraquinone oxidation process, are energy‐intensive and generate toxic byproducts, raising significant environmental and safety concerns.^[^ ^2^ ^]^ This has spurred increasing interest in more sustainable and environmentally friendly approaches for H_2_O_2_ synthesis. Photocatalytic methods, which harness solar energy, water, and oxygen, offer a promising alternative.^[^ ^3^ ^]^ Non‐metallic catalysts, in particular, have gained attention as viable substitutes for metal‐based catalysts due to their potential to efficiently produce H_2_O_2_.^[^ ^4^ ^]^ Additionally, they avoid the drawbacks of metal catalysts, such as the decomposition of H_2_O_2_, thereby enhancing the stability and yield of the product.^[^ ^5^ ^]^

Conjugated organic polymers (CPs) have emerged as promising photocatalysts for H_2_O_2_ synthesis due to their tunable structures and functionalities, which enable precise control over light‐driven processes at the molecular level.^[^ ^6^ ^]^ Despite their potential, the photocatalytic efficiency of organic semiconductors is often limited by insufficient exciton dissociation.^[^ ^7^ ^]^ Recently, many studies have indicated that exciton regulation can effectively enhance charge‐carrier utilization efficiency.^[^ ^8^ ^]^ However, in traditional organic photocatalysts, excitons at room temperature cannot dissociate efficiently to form photogenerated carriers; instead, they undergo spin flip to form singlet oxygen or recombine and dissipate energy as heat.^[^ ^9^ ^]^ Therefore, achieving efficient exciton dissociation at room temperature is crucial for enhancing H_2_O_2_ production efficiency.

To address this challenge, the design of donor‐acceptor (D‐A) polymer structures has been explored.^[^ ^10^ ^]^ By strategically arranging electron donor and acceptor units within these polymers, the exciton activation energy can be lowered, reducing the energy barrier for exciton dissociation. This structural tuning is expected to facilitate exciton separation.^[^ ^11^ ^]^ However, the intrinsic mechanisms behind this process—how these structural modifications promote efficient exciton dissociation—remain poorly understood. Unraveling these underlying mechanisms is a critical area of ongoing research, as they are the key to improving the efficiency of photocatalytic processes.

In this study, we investigate the photocatalytic performance of two conjugated polymer photocatalysts, O‐PTAQ and P‐PTAQ, which share identical building blocks but differ in the substitution sites of the electron donor units. By systematically examining the effects of substitution site modulation, we reveal how enhanced electron delocalization in the electron donor unit lowers exciton activation energy, thereby facilitating exciton separation in O‐PTAQ. Our findings underscore the critical role of structural tuning in improving photocatalytic performance and provide new insights into the design of highly efficient organic photocatalysts for sustainable H_2_O_2_ production.

## 2 Results and Discussion

### 2.1 Design and Characterization of the Photocatalysts

**Figure 1:**
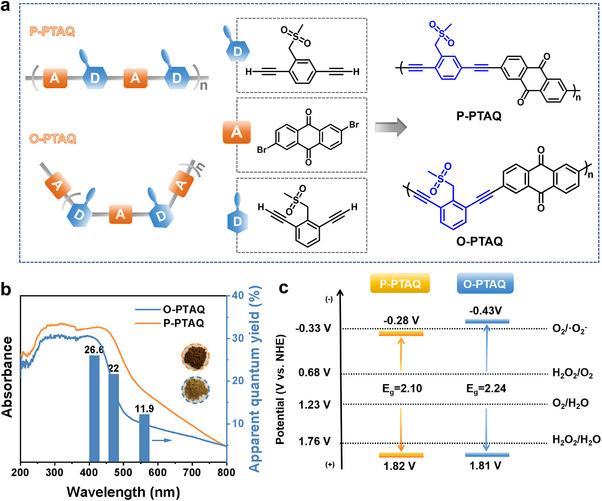
a) Chemical components of catalysts: D represents electron donors; A represents electron acceptor. b) UV–vis diffuse reflectance spectra of the catalysts, along with apparent quantum yield (AQY) at specified wavelengths. c) Schematic illustration of the electronic band structures of the catalysts.

The two conjugated polymers (CPs) were synthesized with 1,3‐PT and 1,4‐PT respectively as electron donors, 2,6‐dibromoanthraquinone (AQ) as electron acceptor, with alkynyl groups serving as the linkers. The resulting photocatalysts were designated as O‐PTAQ and P‐PTAQ respectively (**Figure** **1**a). Among these, the monomers 1,3‐PT and 1,4‐PT were synthesized from commercially available starting materials with satisfactory yields. The synthetic pathways and characterizations are provided in detail in (Schemes S1 and S2, Supporting Information). Both polymers contain the same electron donors, electron acceptors, and linkers, but their D and A are connected at different substitution sites, which leads to different photocatalytic performances of the two conjugated polymers. The identification of electron donors and acceptors is based on their distribution within the highest occupied molecular orbital (HOMO) and the lowest unoccupied molecular orbital (LUMO) of the structure optimized using density functional theory (DFT). As shown in Figure S13 (Supporting Information), the LUMO is predominantly localized on the AQ moiety, while the HOMO is primarily distributed over the benzene ring of the donor unit. This distribution confirms the presence of a donor‐acceptor interaction within the conjugated polymer structure.

The successful polymerization of the samples was determined through various analytical methods. Solid‐state ^13^C‐NMR spectrum showed the alkyne signal peak at ≈90 ppm (Figure S14, Supporting Information), while the Fourier transform infrared (FT‐IR) at ≈2210 cm^−1^ (Figure S16, Supporting Information) and the Raman spectrum at 2200 cm^−1^ (Figure S17, Supporting Information) both corresponded to the stretching vibration of the alkyne group.^[^ ^12^ ^]^ In addition, the ketone carbon signal peak in the ^13^C‐NMR spectrum (≈180 ppm) (Figure S14, Supporting Information), the stretching signal peak of C═O at ≈1670 cm^−1^ in the FT‐IR spectrum (Figure S16, Supporting Information), and the characteristic peak at 287.5 eV in X‐ray photoelectron spectroscopy (Figure S15, Supporting Information), all indicated that the AQ structure was retained in the CPs.^[^ ^13^ ^]^ It was worth noting that the signal peak at 1120 cm^−1^ in the FTIR spectrum was attributed to the stretching vibration peak of the sulfone (O═S═O) group (Figure S16, Supporting Information).^[^ ^14^ ^]^ Combined with the characteristic peak of S 2p shown by XPS (Figure S15, Supporting Information), and the elemental mapping analysis of TEM verified the uniform distribution of S atoms. The above results confirmed the successful introduction of the O═S═O unit into the polymer (Figures S20 and S21, Supporting Information).^[^ ^10a^ ^]^ These results provide substantial confirmation for the successful synthesis of the target photocatalysts. The powder X‐ray diffraction (PXRD) patterns manifested that both photocatalysts displayed the characteristics of amorphous carbon (Figure S18, Supporting Information), signifying typical conjugated polymers.^[^ ^15^ ^]^ Scanning electron microscopy (SEM) and transmission electron microscopy (TEM) analyses revealed that both tested samples exhibited a layered structure, which was in accordance with the expectations for these types of materials (Figures S19–S21, Supporting Information).^[^ ^16^ ^]^ The surface area measured by the Brunell‐Emmett‐Taylor (BET) method disclosed the disparity in surface properties, with P‐PTAQ having a surface area of 87.15 m^2^g^−1^ while O‐PTAQ possessed a larger surface area of 205.26 m^2^g^−1^ (Figure S22 and Table S1, Supporting Information). Thermogravimetric analysis (TGA) indicated that both polymers demonstrated moderate thermal stability, retaining more than 95% of their mass at 250 °C (Figure S23, Supporting Information).

### 2.2 Optical and Electronic Properties of Photocatalysts

Upon elucidating the polymer structures of O‐PTAQ and P‐PTAQ, the light absorption properties and electronic band structures were systematically investigated. The UV–vis diffuse reflectance spectra (UV–vis DRS) of these two conjugated polymers (CPs) revealed that both materials exhibit robust light‐harvesting capabilities within the visible light spectrum (Figure 1b).^[^ ^3c^ ^]^ Analysis via Mott–Schottky plots indicated that both CPs possess n‐type semiconductor characteristics, as evidenced by their positive slopes. Consequently, the conduction band minimum (CBM) values for O‐PTAQ and P‐PTAQ were determined to be −0.43 and −0.28 V versus RHE, respectively (Figure S24, Supporting Information).^[^ ^17^ ^]^ Additionally, the valence band maximum (VBM) positions were accurately established using valence band X‐ray photoelectron spectroscopy (VB‐XPS) and Kelvin probe force microscopy (KPFM) (Figures S25 and S26, Supporting Information).^[^ ^18^ ^]^ Specifically, VB‐XPS was employed to determine the energy level difference between the valence band maximum and the Fermi level, while KPFM measured the potential difference between the Fermi level and the vacuum level, corresponding to the material's work function. By summing these two values and subtracting 4.5 eV, the valence band positions relative to NHE were obtained, which were found to be 1.81 and 1.82 V for O‐PTAQ and P‐PTAQ, respectively. The resulting electronic energy level structures are depicted in Figure 1c, demonstrating that both materials are thermodynamically capable of catalyzing hydrogen peroxide production through the oxygen reduction reaction (ORR) and water oxidation reaction (WOR) pathways.

### 2.3 Photocatalytic Performance

**Figure 2:**
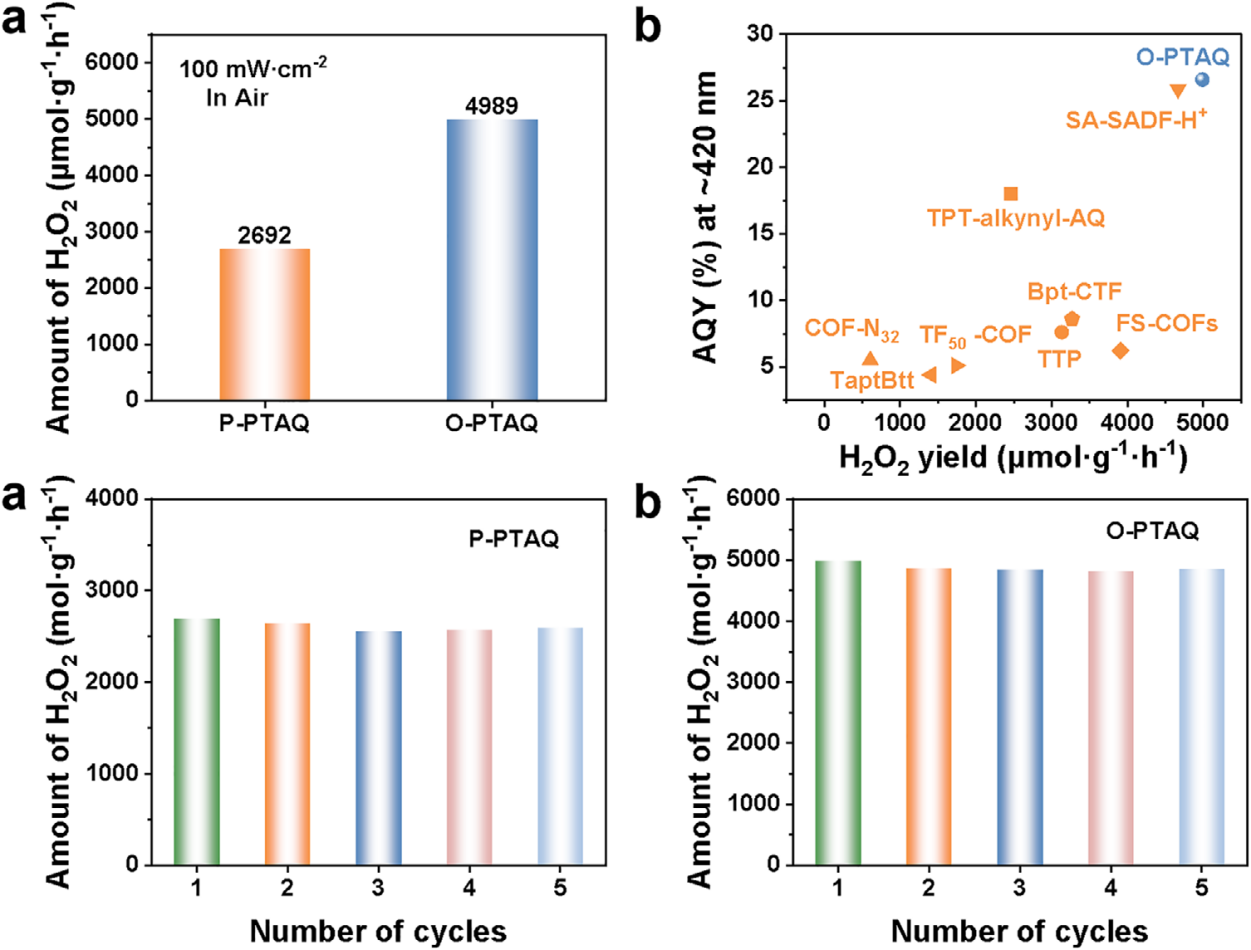
a) Photocatalytic production of H_2_O_2_ by CPs in pure water over 1 h. b) Comparison of the photocatalytic hydrogen peroxide production efficiency and AQY of O‐PTAQ with those of other reported photocatalysts. c) Cycling tests of H_2_O_2_ production on P‐PTAQ in pure water for 5 cycles. d) Cycling tests of H_2_O_2_ production on O‐PTAQ in pure water for 5 cycles.

Subsequently, the performance of CPs in the photocatalytic synthesis of hydrogen peroxide under natural environmental conditions was investigated. As illustrated in **Figure** **2**a, under visible light irradiation (100 mW·cm^−2^), open‐air, and pure water conditions for 1 h, the hydrogen peroxide production rate of O‐PTAQ reached 4989 µmol·g^−1^·h^−1^, significantly surpassing that of P‐PTAQ (2692 µmol·g^−1^·h^−1^) and most organic photocatalysts (Figure 2b; Table S2, Supporting Information). Furthermore, O‐PTAQ demonstrated a high apparent quantum yield (AQY) of 26.6% at 420 nm, markedly higher than the 10.2% observed for P‐PTAQ (Figure 1b). Additionally, the AQY values of O‐PTAQ at wavelengths of 470, 550, and 620 nm were 22%, 11.9%, and 5.2%, respectively (Figure S27, Supporting Information).^[^ ^6b^ ^]^ Moreover, the solar‐to‐chemical energy conversion efficiency (SCC) of O‐PTAQ was 1.64%, superior to most photocatalysts reported in the literature and exceeding the photosynthetic efficiency (≈1%) of most natural plants (Table S3, Supporting Information).^[^ ^16^ ^]^

In addition to demonstrating excellent hydrogen peroxide generation activity, the two conjugated polymers (CPs) maintained superior hydrogen peroxide generation performance over five consecutive cycles (Figure 2c,d), with no significant changes observed in the XRD and FT‐IR spectra before and after cycling. This suggests that under visible light irradiation, the catalysts exhibit excellent photostability (Figure S28, Supporting Information). To evaluate whether CPs could sustain high hydrogen peroxide photocatalytic generation performance in real water samples, the photocatalytic hydrogen peroxide generation rates of the two catalysts were tested in lake water and Pearl River water (Figure S29, Supporting Information). Unlike the efficient hydrogen peroxide synthesis observed in ultrapure water, the hydrogen peroxide synthesis rates in river water and Pearl River water showed a slight decrease after 1 hour of illumination. Specifically, the hydrogen peroxide production rates of O‐PTAQ in lake water and Pearl River water were 4366 and 4318 µmol·g^−1^·h^−1^, respectively. For P‐PTAQ, the corresponding rates in river water and Pearl River water were 1952 and 2076 µmol·g^−1^·h^−1^, respectively. These findings suggest that the catalysts exhibit outstanding hydrogen peroxide synthesis efficiency and have the potential for practical utilization in natural water bodies.^[^ ^14^, ^19^ ^]^

### 2.4 Pathways for H_2_O_2_ Generation

**Figure 3:**
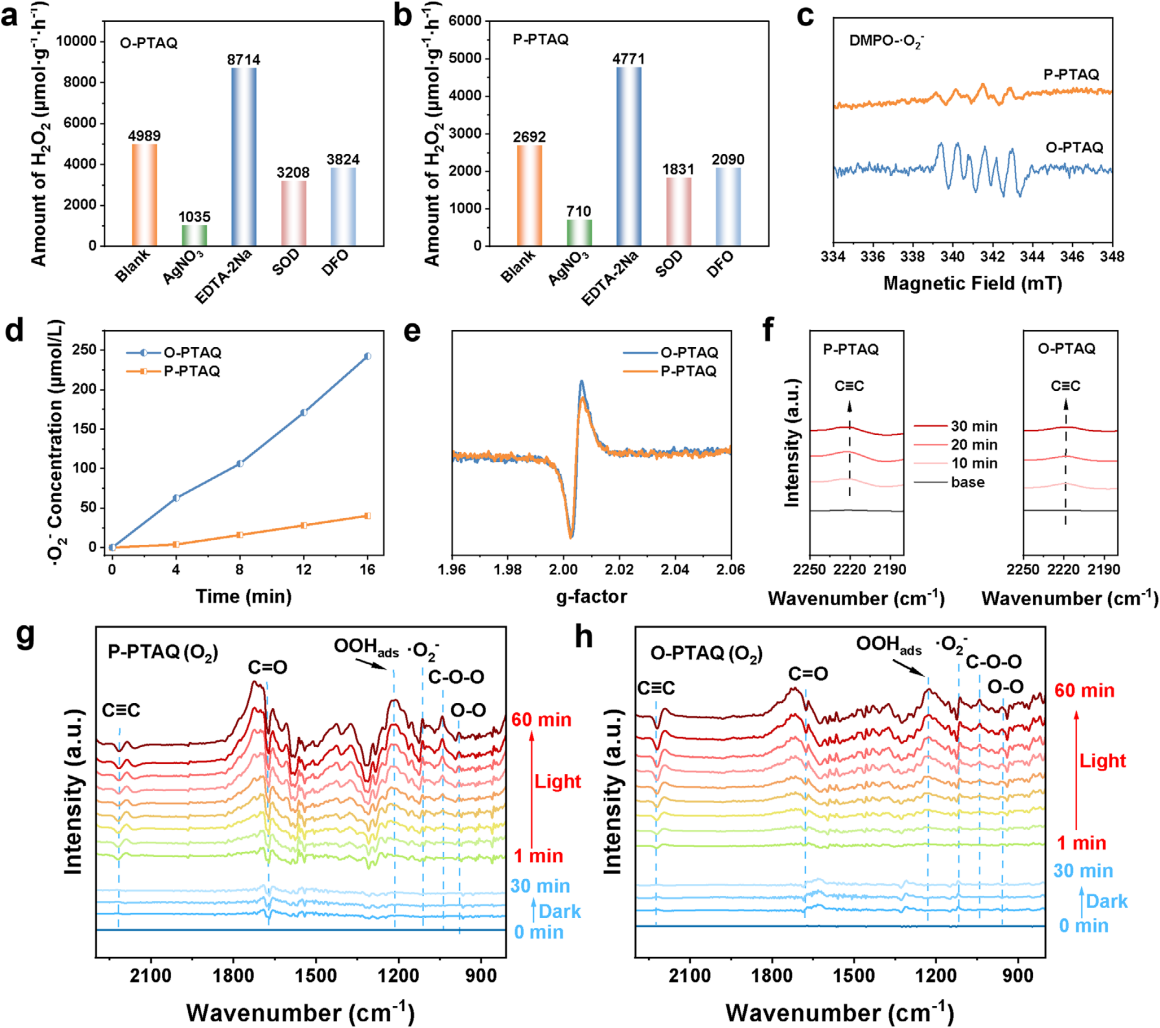
a,b) Photocatalytic H_2_O_2_ production under different control conditions. Reaction conditions: [photocatalyst] = 0.01 g L^−1^, [AgNO_3_] = [EDTA‐2Na] = [SOD] = [DFO] = 10 mm. c) EPR spectra for the detection of ·O_2_^−^ in the presence of DMPO under illumination. d) Concentration of ·O_2_^−^ generated by CPs. e) In situ solid‐state EPR spectra for the detection of OCORs in CPs. f) In situ DRIFTS investigation of oxygen adsorption in the dark for CPs. g,h) In situ DRIFTS of P‐PTAQ and O‐PTAQ under O_2_.

To elucidate the generation pathways of hydrogen peroxide by the two CPs under visible light irradiation, a series of comparative experiments were conducted. Initially, after purging argon gas in pure water to eliminate dissolved oxygen, the hydrogen peroxide yields of O‐PTAQ and P‐PTAQ decreased by 95% and 97%, respectively, indicating the critical role of the oxygen reduction reaction (ORR) in the hydrogen peroxide generation process (Figure S30, Supporting Information).^[^ ^20^ ^]^ Subsequently, radical scavenging experiments were performed. Under argon purging (removing O_2_) and adding silver nitrate (AgNO_3_) as an electron sacrificial agent to the reaction system, hydrogen peroxide could only be generated directly through water oxidation and not via the oxygen reduction pathway.^[^ ^21^ ^]^ The hydrogen peroxide yields of O‐PTAQ and P‐PTAQ decreased by 79% and 74%, respectively (**Figure** **3**a,b). To further investigate the contribution of the WOR pathway, we conducted H_2_^18^O isotope‐labeling experiments. As shown in Figure S31 (Supporting Information), after light irradiation, ^18^O was detected in both O‐PTAQ and P‐PTAQ systems. Notably, the ^18^O content was relatively low, suggesting that the contribution of the WOR pathway to hydrogen peroxide generation is limited. Conversely, adding EDTA disodium (EDTA‐2Na) as a hole sacrificial agent to the reaction system increased the hydrogen peroxide yields by 75% and 77%, respectively, confirming that the ORR is the predominant pathway for hydrogen peroxide generation.^[^ ^12^ ^]^ Analysis of the linear sweep voltammetry (LSV) curves at different rotational speeds of the rotating disk electrode (RDE) revealed that the average electron transfer numbers for ORR on O‐PTAQ and P‐PTAQ were 2.23 and 2.19, respectively, as determined by Koutecky‐Levich plots fitting (Figures S32 and S33, Supporting Information).^[^ ^22^ ^]^ The results demonstrate that oxygen can be selectively reduced to hydrogen peroxide through highly selective two‐electron ORR instead of being fully reduced to water through four‐electron ORR.^[^ ^20^ ^]^

Subsequently, superoxide dismutase (SOD), a scavenger of superoxide radicals (·O_2_^−^), was respectively introduced into O‐PTAQ and P‐PTAQ. As depicted in Figure 3a,b, the hydrogen peroxide yields from O‐PTAQ and P‐PTAQ decreased by 36% and 32%, respectively, indicating that ·O_2_^−^ plays a crucial role as an intermediate in hydrogen peroxide generation.^[^ ^23^ ^]^ To further explore the production of superoxide radicals, electron paramagnetic resonance (EPR) tests were carried out on both CPs, using DMPO as a spin trap for ·O_2_^−^. The results disclosed characteristic DMPO‐·O_2_^−^ signals for both CPs, confirming the presence of superoxide radicals (Figure 3c).^[^ ^24^ ^]^ Notably, the signal intensity of ·O_2_^−^ from O‐PTAQ was significantly higher than that of P‐PTAQ, suggesting that O‐PTAQ generates a larger amount of ·O_2_^−^. Quantitative analysis of ·O_2_^−^ was conducted using nitro blue tetrazolium (NBT). After 16 min of irradiation, O‐PTAQ produced 6.1 times more ·O_2_^−^ than P‐PTAQ (Figure 3d; Figures S34 and S35, Supporting Information). This enhanced production of ·O_2_^−^ intermediates facilitates hydrogen peroxide generation via the ORR pathway, resulting in a significantly higher H_2_O_2_ photosynthetic rate for O‐PTAQ compared to P‐PTAQ.^[^ ^25^ ^]^ Furthermore, the in situ EPR tests of solid powder revealed that signals of oxygen‐centered radicals (OCORs) with a g‐factor of 2.008 were present in both CPs (Figure 3e), suggesting the existence of OCORs.^[^ ^13^ ^]^ When deferoxamine mesylate (DFO), a scavenger of OCORs, was introduced into both reaction systems, the hydrogen peroxide production by O‐PTAQ and P‐PTAQ decreased by 23% and 22%, respectively (Figure 3a,b). This confirms that OCORs serve as an additional intermediate in the generation of hydrogen peroxide.^[^ ^12^ ^]^ To sum up, both O‐PTAQ and P‐PTAQ produce hydrogen peroxide mainly through the ORR pathway, and both include two pathways with superoxide radicals and OCORs as intermediates.

After clarifying the generation pathways and intermediates of hydrogen peroxide, an in‐depth exploration was conducted into the generation sites of these intermediates. In the conventional route for superoxide radical generation, oxygen initially adsorbs onto electron‐deficient functional groups on the catalyst surface and subsequently acquires photogenerated electrons. To investigate the adsorption sites of oxygen, DFT was employed to calculate the oxygen adsorption energy at the active sites of the two catalysts.^[^ ^26^ ^]^ As depicted in Figures S36 and S37 (Supporting Information), active site 1 in O‐PTAQ displayed the lowest oxygen adsorption energy (E_ads_ = −0.139 eV), which was significantly lower than that of sites 2 (E_ads_ = 0.059 eV) and 3 (E_ads_ = 0.059 eV). This implies that the C≡C position is most likely the principal adsorption site for oxygen in O‐PTAQ and thus the probable generation site of superoxide radicals. Similarly, in P‐PTAQ, computational results consistently demonstrated that the C≡C site (E_ads_ = −0.037 eV) presented the lowest oxygen adsorption energy among all the considered sites.

Subsequently, the adsorption sites of oxygen were further verified through in situ diffuse reflectance infrared Fourier transform spectroscopy (DRIFTS). The DRIFTS data revealed that when oxygen was introduced in the dark, the alkyne peaks (≈2200 cm^−1^) of both CPs increased significantly after the introduction of oxygen, confirming the occurrence of oxygen adsorption on the alkyne groups (Figure 3f). Subsequently, upon continuous oxygen introduction during irradiation, the alkyne peaks transformed from upward to downward, and the downward peaks gradually intensified. Simultaneously, the intermediates attributed to the O─O bond, ·O_2_^−^, and ^*^OOH at ≈979, 1113, and 1220 cm^−1^ rose as the alkyne peaks (≈2213 cm^−1^) that adsorbed oxygen declined. This further substantiated that the alkyne groups were the primary generation sites of superoxide radicals for the two photocatalysts (Figure 3g,h).^[^ ^17^ ^]^

The results of the solid EPR test indicated that the signal intensity of OCORs in O‐PTAQ was marginally higher than that in P‐PTAQ. Hence, we inferred that the photogenerated electrons in O‐PTAQ were more inclined to transfer to the electron‐accepting anthraquinone groups (Figure 3e).

**Figure 4:**
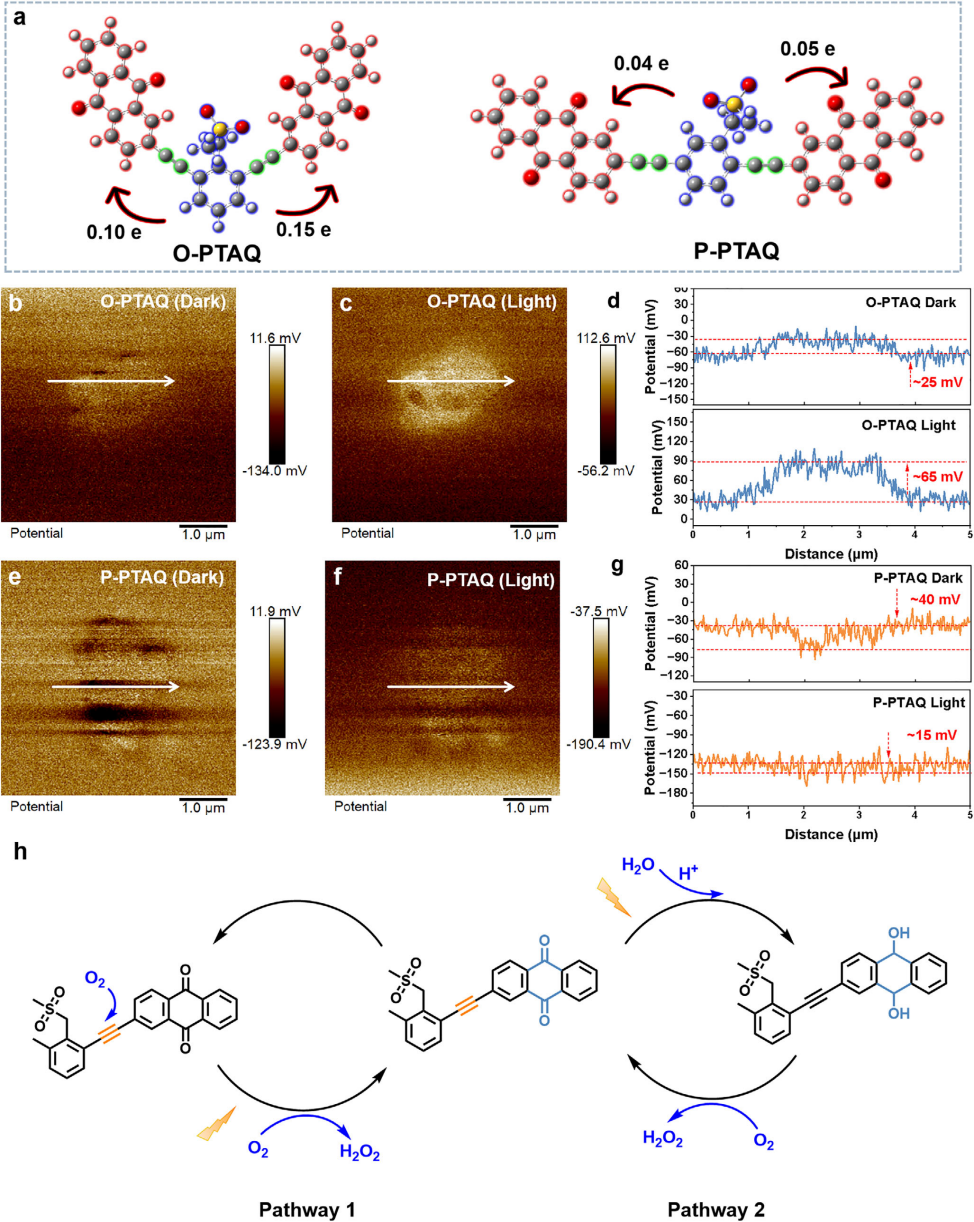
a) Quantitative charge transfer from electron donor to acceptor. b–f) In situ KPFM image. d,g) corresponding potential profile along 1 to 5 µm of CPs. h) The proposed pathways of photosynthetic H_2_O_2_ production.

Quantitative analysis of the charge transfer from the electron donor to the electron acceptor by DFT disclosed that, compared with 0.09 e in P‐PTAQ, 0.25 e in O‐PTAQ provided more electrons to the acceptor anthraquinone, which also confirmed that the substitution site where D and A were connected in O‐PTAQ was more conducive to intramolecular electron transfer (**Figure** **4**a).^[^ ^12^ ^]^ To further substantiate the intramolecular charge transfer in CPs, we performed an in situ Kelvin probe force microscopy (KPFM) analysis (Figure 4b–g). Specifically, by quantitatively assessing the variations in surface potential before and after illumination, we directly examined the characteristics of charge redistribution. During the KPFM measurements, distinct differences were observed in the potential change behaviors of O‐PTAQ and P‐PTAQ upon illumination. More specifically, the potential difference between the highest potential point and the baseline potential for O‐PTAQ increased from ≈25 to ≈65 mV, yielding a total potential difference of ≈40 mV. This value is considerably higher than the ≈25 mV measured for P‐PTAQ. This pronounced potential variation suggests that a more efficient charge separation and transfer process occurs within O‐PTAQ under photoexcitation conditions, indicating its superior intramolecular charge transfer efficiency compared to P‐PTAQ.^[^ ^27^ ^]^ Additionally, the reaction sites of OCORs were also disclosed through in situ infrared spectroscopy. The C═O peak (≈1675 cm^−1^) at the electron acceptor anthraquinone presented slight variations before and after irradiation in DRIFTS, indicating that the C═O bond of anthraquinone was the principal generation site of OCORs (Figure 3g,h). This result was in accordance with the EPR results, indicating that oxygen‐centered organic radicals were generated on the anthraquinone groups in both catalysts. Therefore, in O‐PTAQ, the alkyne group was the generation site of superoxide radicals, and anthraquinone was the generation site of OCORs (Figure 4h).^[^ ^22^ ^]^

### 2.5 Mechanism Underlying the High Efficiency of O‐PTAQ

The generation processes of both superoxide radicals and OCORs necessitate the involvement of photogenerated electrons. Specifically, in the case of superoxide radicals, oxygen acquires the photogenerated electrons, and for OCORs, AQ acquires the photogenerated electrons. Therefore, the quantity of photogenerated electrons is crucial for the generation of these two intermediates and the final production quantity of hydrogen peroxide. Hence, we undertook a more profound exploration of the influence of substitution sites on the generation quantity of photogenerated electrons in these two catalysts. First, the transient photocurrent response test was conducted in the saturated Ar solution. The results suggested that O‐PTAQ had a higher photocurrent density than P‐PTAQ, ≈3 times as high as that of P‐PTAQ. Moreover, the difference in photocurrent between O‐PTAQ and P‐PTAQ became more pronounced in the presence of oxygen (Figure S38, Supporting Information). This is because, compared with the argon environment, O‐PTAQ in the oxygen environment can more effectively utilize the photogenerated electrons to react with oxygen.^[^ ^27^, ^28^ ^]^ This process enhances the separation and transport of photogenerated carriers, reduces the recombination of carriers, and significantly increases the photocurrent. This further indicates that in the photocatalytic process, O‐PTAQ would generate a larger number of photogenerated charge carriers than P‐PTAQ.^[^ ^29^ ^]^

**Figure 5:**
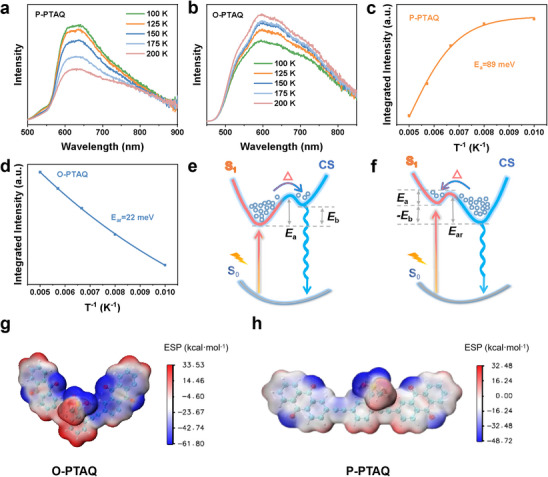
a,b) Temperature‐dependent photoluminescence (PL) spectra for P‐PTAQ and O‐PTAQ. c,d) Evolution of PL intensity as a function of temperature from 100 to 200 K for P‐PTAQ and O‐PTAQ. e) Illustration of mutual transitions between the charge‐separated state (CS) and the lowest singlet excited state (S_1_) in the common materials in which the value of exciton binding energy (E_b_) is positive and E_a_ is the activation energy from S_1_ to CS. f) Illustration of mutual transitions between CS and S_1_ in O‐PTAQ, in which the value of E_b_ is negative and E_ar_ is the activation energy from CS to S_1_. g,h) Electrostatic potential (ESP) of CPs.

To explore the reasons for the generation of more photogenerated charge carriers in O‐PTAQ, the activation energy of exciton dissociation (E_a_) or activation energy of charge recombination (E_ar_) was determined through the temperature‐dependent photoluminescence (PL) spectra.^[^ ^30^ ^]^ It can be observed from **Figure** **5**a that the PL intensity of P‐PTAQ decreased with the increase in temperature within the temperature range from 100 to 200 K. In sharp contrast, the PL intensity of O‐PTAQ increased with the rise of temperature (Figure 5b). This indicates that at room temperature for O‐PTAQ, the energy barrier for excitons to dissociate into free charges is lower than the thermal energy, and the energy level of the charge separation state is lower than that of the exciton state (Figure 5e,f), that is, E_a_ is less than E_ar_, and the exciton binding energy (E_b_) is negative. This implies that for O‐PTAQ at room temperature, the dissociation of excitons is spontaneous.^[^ ^18^ ^]^ The variation law of fluorescence intensity with respect to temperature was fitted through the utilization of the Arrhenius Equation (1):

(1) $$I\left(T\right)=\frac{I_{0}}{1+A\cdot \exp\left(\frac{-E_{a}}{k_{B}T}\right)}\;$$

where *I*(*T*) represents the integrated PL intensity and *k*_B_ is the Boltzmann constant. *A* and *E*_a_ were obtained by fitting the temperature‐intensity diagram.

As shown in Figure 5c, the E_a_ for P‐PTAQ was 89 meV, while the increase of PL of O‐PTAQ with temperature indicated that E_a_ was less than the reverse barrier E_ar_. Since the reverse temperature‐dependent PL intensity was 22 meV, only E_ar_ of O‐PTAQ could be estimated. Therefore, the E_a_ of O‐PTAQ should have been even less than 22 meV (Figure 5d).^[^ ^7a^ ^]^ These results suggested that the spontaneous separation energy of O‐PTAQ promoted the more efficient conversion of excitons into photogenerated carriers, thereby improving its photocatalytic performance.

To further explore the mechanism underlying the spontaneous dissociation of excitons, we performed DFT calculations. The internal charge separation (Pi) and molecular polarity index (MPI) of the two catalysts are presented in Table S4 (Supporting Information). The Pi and MPI of O‐PTAQ were 15.11 and 14.95, respectively, higher than those of P‐PTAQ (13.12 and 13.21).^[^ ^31^ ^]^ Pi serves as a quantitative indicator of the extent of charge separation within a system, reflecting the efficiency of this process. A higher Pi value indicates more effective charge separation within the material. The Molecular Polarity Index (MPI) serves as an indicator representing the degree of molecular polarity. The larger the MPI, the greater the overall polarity of the molecule. This is because the uneven charge distribution in the system reflects the polarity of the molecule. As a result, there are more positive and negative regions of electrostatic potential on the molecular surface. Further, it can be observed from the electrostatic potential diagrams in Figure 5g,h that the electrostatic potential range of O‐PTAQ is from −61.80446 to 33.53088 kcal·mol^−1^, while the range of P‐PTAQ is from −48.71849 to 32.48205 kcal·mol^−1^. The broader electrostatic potential distribution range confirms that the O‐PTAQ material has stronger charge delocalization and charge separation ability than P‐PTAQ.^[^ ^32^ ^]^ The above evidence demonstrates that the strong delocalization of O‐PTAQ is the fundamental reason for the rapid generation of polarons. The enhancement of O‐PTAQ's overall delocalization can be attributed to the substitution of electron donor units at the 1,3 positions. As illustrated in Figure S39 (Supporting Information), compared to 1,4‐PT, the 1,3‐PT monomer exhibits a broader distribution of electrostatic potential, confirming its superior charge delocalization.^[^ ^33^ ^]^ We calculated the Mulliken electronegativity of the monomer. The smaller the Mulliken electronegativity, the stronger the electron‐giving ability.^[^ ^31b^ ^]^ As shown in Figure S40 and Table S5 (Supporting Information), the 1,3‐PT monomer is more capable of giving electrons than the 1,4‐PT monomer, while the AQ monomer tends to gain electrons. When the electron‐withdrawing AQ group is substituted at the 1,3 positions, it lowers the electron density at the electron donor center, resulting in a more pronounced electron cloud delocalization compared to the 1,4 substitution positions in P‐PTAQ. This further promotes the efficient dissociation of excitons and enhances the material's overall electronic properties.^[^ ^34^ ^]^

**Figure 6:**
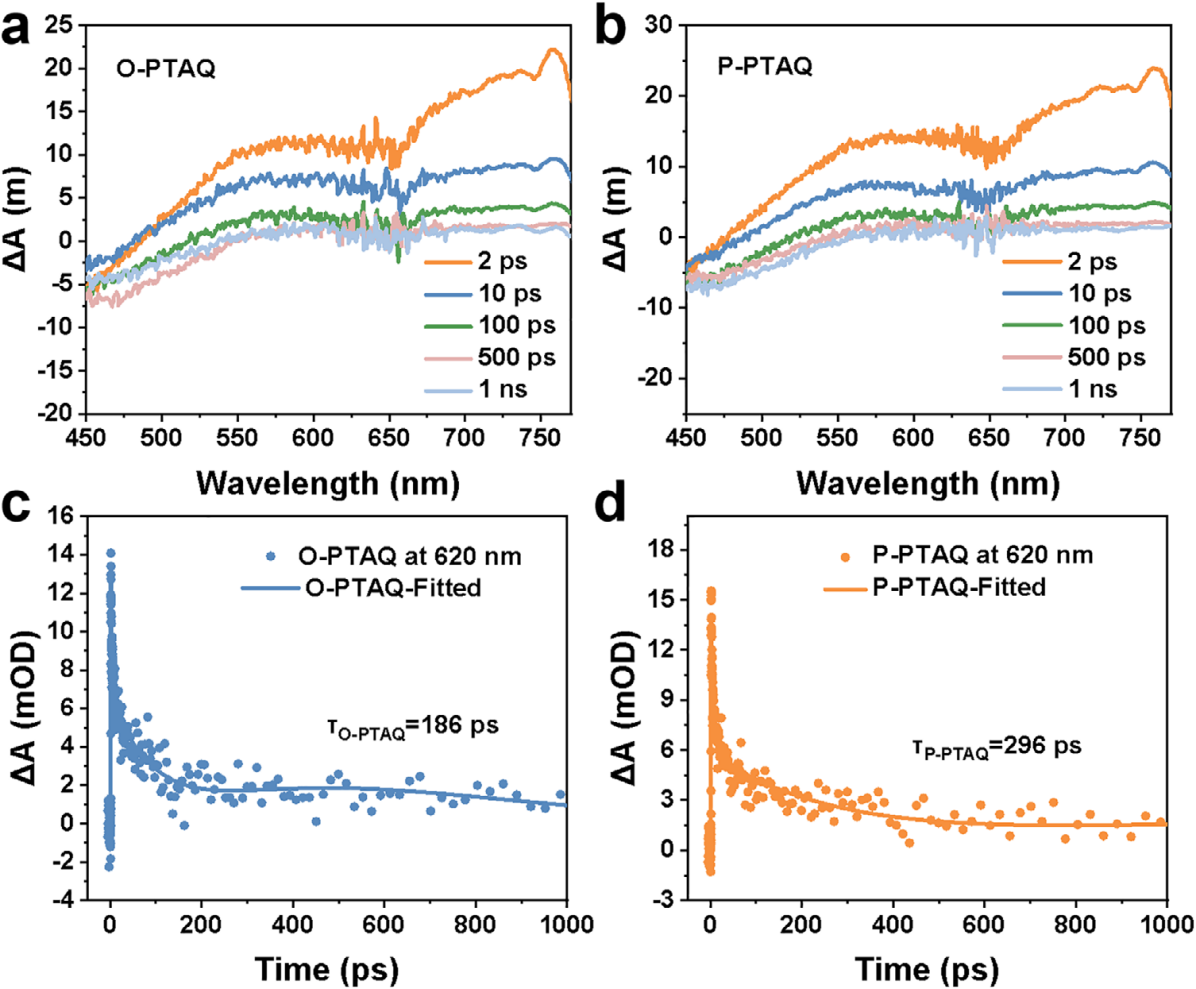
a,b) TA spectra of CPs pumped at 400 nm in water. c,d) TA kinetics of O‐PTAQ and P‐PTAQ probed at 620 nm.

Additionally, regarding correlating the substitution site with exciton dissociation dynamics, transient absorption (TA) spectroscopy can be used to measure the time required for exciton dissociation. The exciton dissociation process involves the dissociation of excitons to form polarons, and then the polarons are converted into free electrons. The time from excitons to polarons is on the femtosecond scale, while the time from polarons to free electrons is on the picosecond scale. Therefore, we focused on studying the time required for polaron dissociation.^[^ ^35^ ^]^ As shown in **Figure** **6**a,b, both catalysts displayed broad positive peaks within the range of 500–650 nm. To ascribe this peak, we separately added AgNO_3_ as the electron sacrificial agent and EDTA‐2Na as the hole sacrificial agent in the system and observed the alterations of the peak under the same test conditions. It was discovered that compared with the solution without adding scavengers, the peak intensity was notably reduced in the presence of AgNO_3_ or EDTA‐2Na, indicating that accelerating the reaction of either electrons or holes could accelerate the decay rate of this peak, which was in line with the nature of polarons. Therefore, this peak was attributed to the polaron peak (Figure S41, Supporting Information).^[^ ^17^ ^]^ Further analysis of the peak at 620 nm revealed that the polaron lifetimes of O‐PTAQ and P‐PTAQ were at the picosecond level and were 186 and 296 ps, respectively (Figure 6c,d). This outcome provides direct evidence that lower exciton activation energy accelerates exciton dissociation.

Furthermore, O‐PTAQ presented a smaller semicircular radius in electrochemical impedance spectroscopy (EIS), indicating a lower internal resistance and relatively easier charge transfer in contrast to P‐PTAQ (Figure S42, Supporting Information).^[^ ^36^ ^]^ To summarize, compared to P‐PTAQ, the higher hydrogen peroxide synthesis efficiency of O‐PTAQ can be ascribed to more photogenerated charge carriers, and the greater generation of photocarriers can be attributed to the spontaneous separation of excitons, a faster exciton‐to‐polaron conversion rate, and faster charge transfer.

## 3 Conclusion

This work reveals a mechanism for reducing exciton activation energy to enhance photocatalytic H_2_O_2_ production. By strategically substituting electron donor units at different positions, we show that increasing the delocalization of the electron cloud in the electron donors enhances the overall material's delocalization. This, in turn, effectively lowers the exciton activation energy, promoting spontaneous exciton dissociation at room temperature. As a result, more excitons activate and dissociate more rapidly, leading to increased generation of superoxide radicals (·O_2_^−^) and oxygen‐centered organic radicals (OCORs), thereby significantly enhancing the H_2_O_2_ production rate. Under room temperature, visible light, in pure water, and without sacrificial agents, O‐PTAQ achieves a photocatalytic H_2_O_2_ synthesis rate of 4989 µmol·g^−1^·h^−1^. Additionally, the solar‐to‐chemical energy conversion efficiency (SCC) of O‐PTAQ reaches 1.64%, surpassing most reported photocatalysts and exceeding the photosynthetic efficiency (≈1%) of natural plants. This work provides new insights into the design of photocatalysts for sustainable H_2_O_2_ production, highlighting the crucial role of reducing exciton activation energy in promoting efficient exciton dissociation.

## Conflict of Interest

The authors declare no conflict of interest.

## Supporting information



Supporting Information

## Data Availability

The data that support the findings of this study are available in the supplementary material of this article.
