# Supplementary material for: Revealing the Mechanism of Exciton Spontaneous Separation at Room Temperature for Efficient Photocatalytic Hydrogen Peroxide Synthesis
Source: Adv Sci (Weinh). 2025 May 19;12(30):e03929. doi: 10.1002/advs.202503929 (PMC12376678; doi:10.1002/advs.202503929)
Supplement: Supplementary file 1 — Supporting Information [file ADVS-12-e03929-s001.docx]

***Supporting Information***

**Revealing the Mechanism of Exciton Spontaneous Separation at Room Temperature for Efficient Photocatalytic Hydrogen Peroxide Synthesis**

Pan Jiang,^[a]^ Yuyan Huang,^[b]^ Xiangqiong Jiang,^[b]^ Huijie Yan,^[a]^ Shufang Liu,^[a]^ Zuoming Chen,^[a]^ Xin Wu,^[a]^ Xiantai Zhou,^[a]^ Yu-Xin Ye*^[a][c]^ and Gangfeng Ouyang*^[a][b][c]^

[a] P. Jiang, H. Yan, S. Liu, Z. Chen, Dr. X. Wu, Dr. X. Zhou, Dr. Y. -X. Ye, Prof. G. Ouyang
School of Chemical Engineering and Technology, ICGME
Sun Yat-sen University
Zhuhai 519082 (P. R. China)
E-mail: [yeyuxin5@sysu.edu.cn](mailto:yeyuxin5@sysu.edu.cn); cesoygf@mail.sysu.edu.cn

[b] Y. Huang, X. Jiang, Prof. G. Ouyang
Key Laboratory of Bioinorganic and Synthetic Chemistry of Ministry of Education, LIFM, School of Chemistry, IGCME
Sun Yat-sen University
Guangzhou 510275 (P. R. China)

[c] Dr. Y. -X. Ye, Prof. G. Ouyang
Southern Marine Science and Engineering Guangdong Laboratory
(Zhuhai)
Zhuhai, Guangdong 519082 (P. R. China)

# Experimental section

## Materials

N-bromosuccinimide (NBS, 99%) and sodium methanesulfinate (95%) were purchased from Aladdin Industrial Corporation and used as received. 2,5-Dibromotoluene (98%), 2,6-Dibromotoluene (98%), 2,6-Dibromoanthraquinone (97%), Cuprous iodide (CuI, 99%), 2,2-azobisisobutyronitrile (AIBN, 98%), Bis(triphenylphosphine)palladium(II) chloride (Pd(PPh_3_)Cl_2_, 98%), (triisopropylsilyl)acetylene (97%), Tetrahydrofuran (THF, 99.9%, Extra Dry), tetrabutylammonium (TBAF, 98%), Piperidine, Acetonitrile (CH_3_CN, 99.9%, Extra Dry) and N, N-dimethylformamide (DMF, 99.8%, Extra Dry) were purchased from Energy Chemical company and used as received. All other reagents and solvents were used directly without further purification. The actual water bodies were sampled from Central Lake and the Pearl River in Guangzhou.

## Characterization

The PXRD data was obtained using the D-MAX 2200 VPC. SEM images were performed on SU8010. TEM images were obtained by JEM-2010. Fourier transform infrared spectroscopy (FT-IR) data was conducted using the Perkinelmer Frontier spectrometer. Solid-state ^13^C NMR spectroscopy was collected using the Bruker AVANCE 600 MHz. X-ray photoelectron spectroscopy (XPS) was measured by Thermo Scientific Nexsa using Al Kα radiation as excitation source. The solid state UV-visible diffuse reflectance spectrum was measured with Shimadzu UV-3600 spectrometer. Photoluminescence (PL) spectra were measured on an FLS 1000 spectrophotometer. Photoelectron chemistry and electrochemical measurements were made using the CHI 760E meter and electrochemical workstation. Electron paramagnetic resonance (EPR) signals are measured on the Bruker A300 spectrometer. In situ diffuse reflection infrared Fourier transform spectroscopy was determined by German Bruker INVENIO S spectrometer.

## Determination of H_2_O_2_ concentration

The concentration of H_2_O_2_ was determined by TMB-H_2_O_2_-HRP method. The reaction of H_2_O_2_ with TMB was as follows：

$$\text{H}_{2}\text{O}_{2}+TMB\overset{\text{HRP}}{\to}\text{H}_{2}\text{O+oxTMB}$$

Preparation of HRP solution: 0.002g horseradish peroxidase (HRP) was dissolved in 10ml deionized water.

Preparation of TMB solution: 0.015 g of 3,3',5,5'-tetramethylbenzidine (TMB) was dissolved in 0.3 mL of DMSO, then add 5 mL glycerol and 45 mL deionized water containing 0.02 g EDTA and 0.095 g citric acid. Finally, 50 mL of deionized water was added to the solution.

The calibration curve was prepared by mixing TMB and HRP with a known concentration of H_2_O_2_ solution, followed by the addition of 10 μL HCl after 3 minutes. The subsequent determination was carried out using UV-vis spectroscopy at 450 nm to establish the linear relationship between signal intensity and H_2_O_2_ concentration. This standard calibration curve was then utilized for the quantitative determination of H_2_O_2_ concentration.

## Photoelectrochemical and electrochemical measurements

All materials were prepared by adding 5 mg of catalyst into the solution containing 180 μL of ethanol and 20 μL of 5% Nafion. The mixture was dispersed through ultrasonication.

All measurements were carried out in a three-electrode cell system of the electrochemical workstation (CHI 760E instrument). The standard three-electrode system comprises a working electrode, Ag/AgCl (saturated KCl) as the reference electrode, and a Pt mesh as the counter electrode. A 300 W Xe lamp was utilized as the illumination source. The mixture was dropped onto FTO glass for Mott-Schottky and photocurrent tests, and both were conducted in 0.1 M sodium sulfate solution. The EIS test was carried out in 0.1 M phosphate buffer solution (pH = 7). The photocurrent measurement was obtained under saturated argon conditions illuminated by a 300 W xenon lamp.

The electrochemical rotating disk electrode (RDE) measurement entails replacing the working electrode with a rotating disk electrode. 5 μL of the catalyst ink was dropped onto the electrode surface and a catalyst film was formed after drying. Linear sweep voltammograms (LSV) were measured at different rotation speeds in O_2_-saturated 0.1 M phosphate buffer solution (pH 7). The corresponding average number of electron transfer was estimated by the Koutecky-Levich equation:

$$\frac{1}{J}=\frac{1}{J_{L}}+\frac{1}{J_{K}}=\frac{1}{B \omega^{1/2}}+\frac{1}{J_{K}}$$

$$B=0.2 nFV^{-1/6}CD^{2/3}$$

Where J represents the measured current density, J_K_ indicates the kinetic current density, J_L_ is the limiting diffusion current density, ω denotes the angular velocity, n is the number of transferred electrons, F stands for the Faraday constant (96485 C∙mol^-1^), V is the kinetic viscosity of water (0.01 cm^2^∙s^-1^), C is the saturated oxygen concentration in the electrolyte (1.26×10^-3^ mol∙L^-1^), and D is the diffusion coefficient of O₂ (2.7 ×10^-5^cm^2^∙s^-1^).

## AQY measurement of H_2_O_2_ Production

The apparent quantum yield (AQY) was measured under a multi-channel light panel furnished with multiple single-wavelength LEDs (CEL-LEDS35). The photocatalytic reaction was conducted by introducing 10 mg of the photocatalyst into a quartz tube containing 50 mL of pure deionized water. The tube was subjected to irradiation from an LED lamp for one hour while being stirred magnetically. The number of incident photons (M) was calculated as follows:

$$M=\frac{E\lambda}{\mathrm{hc}}$$

In the equation, E represents the average irradiation intensity, λ represents the irradiation wavelength, h is the Planck constant, and c represents the speed of light. The calculation equation of quantum efficiency is as follows:

$$\text{AQY=}\frac{2\times\text{number of elvoved H}_{2}\text{O}_{2}\text{ }\text{molecules}}{\text{number of incident photons}}\times100\%$$

## Solar-to-chemical conversion (SCC) efficiency

The SCC efficiency was determined with the photocatalyst (100 mg) dispersed in pure deionized water (100 mL), employing an AM 1.5 G solar simulator as the light source (100 mW∙cm^-2^). The photoreaction was conducted in a glass bottle and the irradiation area was 1 × 10^-4^ m^2^. The SCC efficiency (η) was calculated by the following equation:

$$SCC=\frac{\triangle GH_{2}O_{2}\times nH_{2}O_{2}}{t_{\mathrm{ir}}\times S_{\mathrm{ir}}\times I_{\mathrm{AM}}}\times100\%$$

where ΔGH_2_O_2_ is the free energy for H_2_O_2_ generation (117 kJ mol^-1^, nH_2_O_2_ is the amount of H_2_O_2_ generated, and t_ir_ is the irradiation time (3600 s). I_AM_ and S_ir_ represent the irradiation intensity and area.

## Electron paramagnetic resonance (EPR) measurements

Spin trapping-EPR tests were documented with a Bruker A300 spectrometer. 5,5-dimethyl-1-pyrroline N-oxide (DMPO) was employed as a spin-trapping reagent for the detection of O_2_^•-^. The samples were made by adding 0.5 mg of catalysts into 200 μL of methanol which contained the trapping agents. Light-induced EPR signals were gathered using a 300 W Xenon lamp.

The in-situ EPR tests for the exploration of OCORs were also conducted using the Bruker A300 Electron Paramagnetic Resonance. A certain amount of solid catalysts was placed into a capillary, which was sealed after being purged with argon and exposed to light for a specific period. EPR signals were recorded at fixed intervals.

## TAS Measurements

The TA spectra of CPs were measured using the Helios femtosecond transient absorption spectrometer (Ultrafast Systems, LLC). A 400-nm pump pulse, generated by an optical parametric amplifier (OPerA Solo, Coherent), had a laser intensity of 150 μW. The sample was prepared in the following way: 10 mg of the photocatalyst was dispersed in 20 mL of deionized water. Then the suspension underwent ultrasonic treatment for 36 hours. The initial suspension was centrifuged at 3000 rpm for 10 minutes to eliminate large aggregates. The supernatant was extracted for subsequent detection. To identify the observed signal, electron (AgNO_3_) and hole (EDTA-2Na) sacrificial agents (10 mM) were added to the supernatant.

## Oxidation experiment of NBT

1 mg of the fabricated catalyst was suspended in 50 mL of NBT aqueous solution and exposed to irradiation from a xenon lamp. Prior to irradiation, it was constantly stirred in the darkness for 30 min. Once the reaction concluded, 3 mL of the reaction suspension was obtained. The photocatalyst was isolated through centrifugation, and the absorbance of the supernatant was documented on a UV-Vis spectrophotometer (Shimadzu UV-3600 spectrometer). The photocatalytic production of ·O_2_⁻ was ascertained by the degradation of NBT, which was supervised by the alteration in absorbance at a wavelength of 259 nm.

The superoxide radical produced was calculated according to the following formula:

$$NBT+4O_{2}^{\cdot-}+2H^{+}\to Formazan+4O_{2}$$

## Density Functional Theory (DFT) Calculation

The DFT calculations were conducted in the Gaussian 09 D.01 program package with Grimme-D3 dispersion correction.^[1]^ GaussView5 was employed for visualization.The geometries of simplified fragments of the catalysts were performed by method B3LYP/6-31G**. There are no imaginary frequencies for all optimized structures.^[2, 3]^ Time-dependent density functional theory (TD-DFT) was calculated at pbe1pbe/6-31G (d,p) level of theory and was used to investigate the transfer direction of electrons. Multiwfn was used for hole-electron analysis.^[4]^ The optimized structures were utilized to calculate single-point energy with b3lyp/def2TZVP.The electrostatic potential and Mulliken electronegativity involved in the analysis were evaluated by Multiwfn based on an efficient algorithm and filled with colors using VMD1.9.3^[5]^.

The adsorption energy (E_adsorb_) of O_2_ molecule on the surface is calculated as follow:

| E_adsorb_ = E_total_ - E_surface_ - E_O₂_ |  |  |
| --- | --- | --- |

where E_total_ represents the energy of surface with adsorbed O_2_ molecule, E_surface_ and E_O₂_ represent the energies of isolated surface and O_2_, respectively.

# Synthesis route of diyne monomers

# **2.1** Synthesis of 1,4-P, 1,4-PT–TIPS, 1,4-PT

**Scheme S1.** Synthetic routes of the **1,4-P, 1,4-PT–TIPS** and **1,4-PT**.

(1): S*ynthesis of 1, 4-dibromo-2-(bromomethyl) benzene*

2,5-dibromotoluene (10.3 g, 41.21 mmol), NBS (8.8 g, 49.45 mmol), and AIBN (0.34 g, 8.24 mmol) were dissolved in 120 ml of acetonitrile. The mixture was heated to 85 °C under a nitrogen atmosphere and refluxed for 3 hours. After cooling to room temperature, the sample was dried by rotary evaporation. The product was obtained by chromatography on a column of petroleum ether, yielding 7.45 g of white crystals with a yield of 55.0%.^[6]^

(2): *Synthesis of 1,4-dibromo-2-((methylsulfonyl) methyl) benzene (****1,4-P****)*

1,4-dibromo-2-(bromomethyl) benzene (5 g, 15.3 mmol) and sodium methanesulfinate (4.7 g, 46.0 mmol) were added to DMF (50 mL) and reacted at 70°C for 3 hours. It was cooled to room temperature, settled in saturated NaCI aqueous solution, filtered and dried to obtain 4.8g white product with 96.2% yield.^[6]^

^1^H NMR (400 MHz, DMSO-*d*_6_) δ 7.74 (d, J = 2.4 Hz, 1H), 7.68 (d, J =8.6 Hz, 1H), 7.55（dd, J = 8.5, 2.5 Hz, 1H), 4.67 (s, 2H), 3.04 (s, 3H). ^13^C NMR (101 MHz, DMSO-*d*_6_) δ 136.02, 135.33, 133.73, 131.71, 124.80, 121.01, 58.93, 41.21. *m/z*: [M]^+^ calcd for C_8_H_8_Br_2_O_2_S, 328.02; found 351.03.


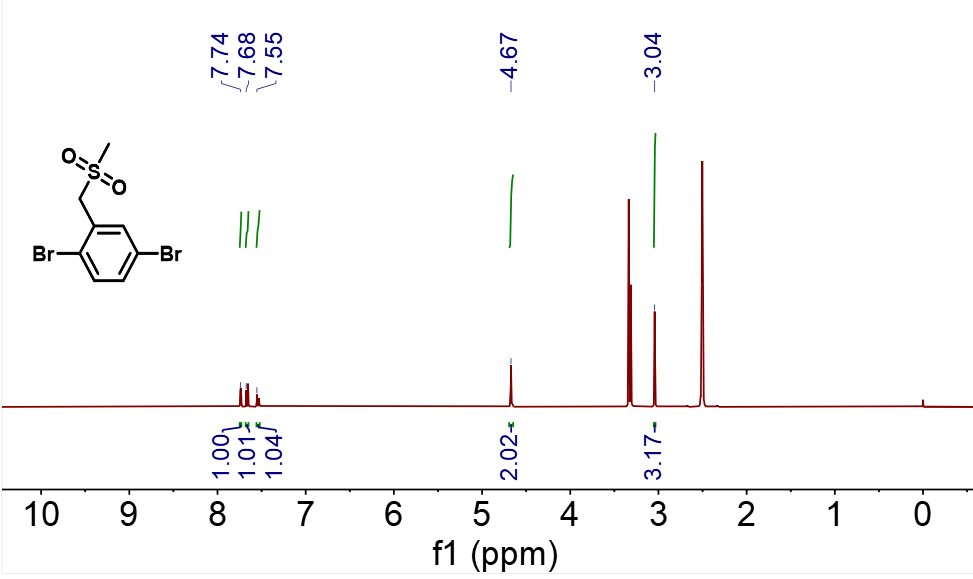


**Figure S1**. ^1^H NMR (400 MHz, DMSO-*d*_6_, 298 K) spectrum of **1,4-P**.


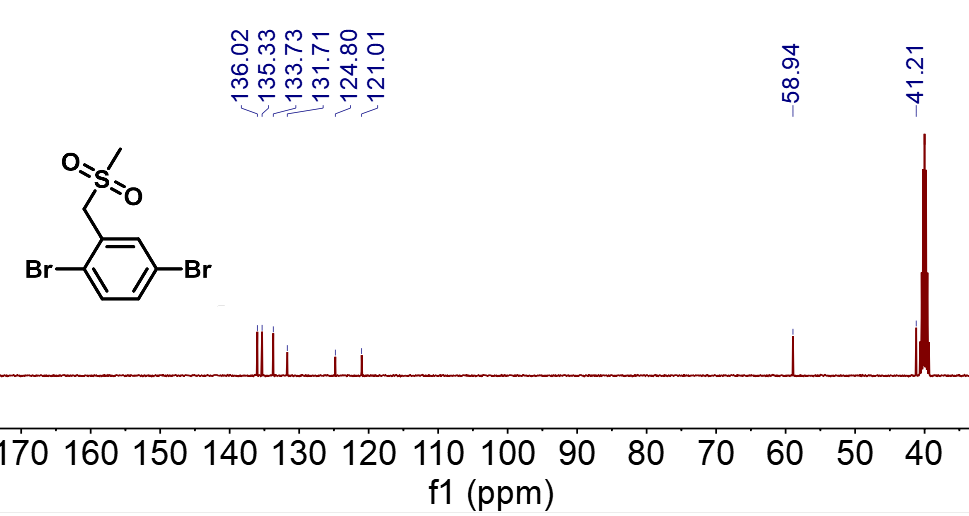


**Figure S2**. ^13^C NMR (101 MHz, DMSO-*d*_6_, 298 K) spectrum of **1,4-P**.

(3): *Synthesis of ((2-((methylsulfonyl)methyl)-1,4-phenylene)bis(ethyne-2,1-diyl))bis(triisopropylsilane) (****1,4-PT–TIPS)***

1,4-dibromo-2-((methylsulfonyl) methyl) benzene (1.058 g, 3.2mmol), triisopropyl silyl acetylene (1.411 g, 8.1 mmol), bistriphenylphosphine palladium dichloride (22 mg, 0.3 mmol), cuprous iodide (61 mg, 0.32 mmol) was dissolved in piperidine (100 ml), heated to 70℃ for 24 h under the protection of argon, and then spin dried at room temperature. 1.34g light yellow solid was obtained by dichloromethane column chromatography with a yield of 93%.

^1^H NMR (400 MHz, DMSO-*d*_6_) δ 7.61 (d, J = 2.4 Hz, 1H), 7.53 (d, J =8 Hz, 1H), 7.48（d, J = 8 Hz, 1H), 4.65 (s, 2H), 2.91 (s, 3H). 1.10 (s, 42H). ^13^C NMR (101 MHz, DMSO-*d*_6_) δ 134.77, 133.49, 132.28, 131.65, 124.60, 123.22, 106.42, 104.53, 97.87, 93.42, 58.10, 18.90, 11.16. m/z: [M]^+^ calcd for C_30_H_50_O_2_SSi_2_, 530.96; found 553.30.


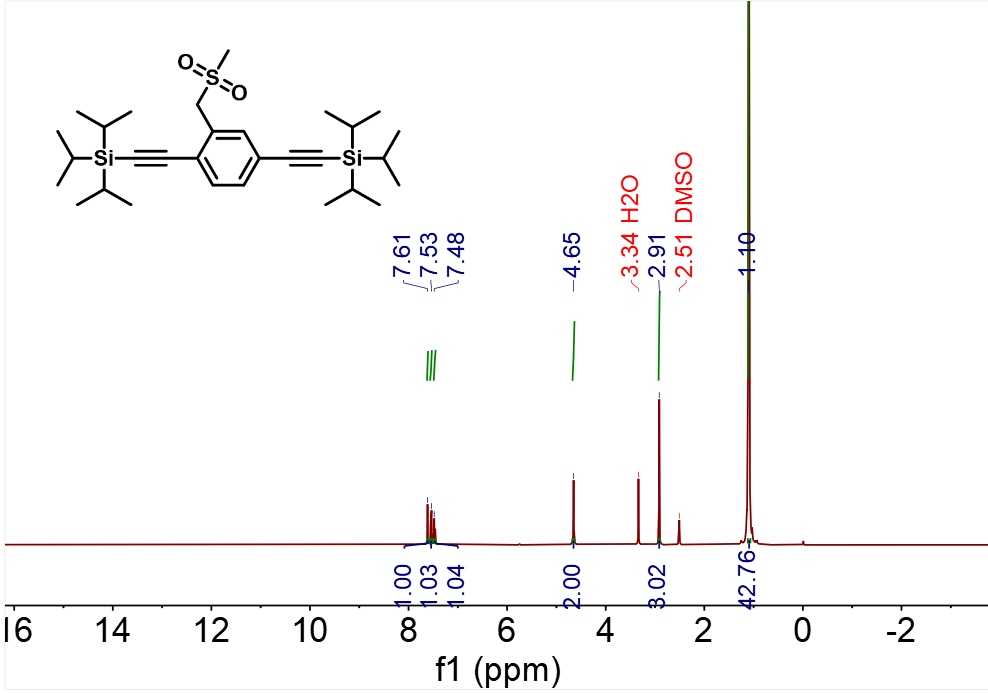


**Figure S3**. ^1^H NMR (400 MHz, DMSO-*d*_6_, 298 K) spectrum of **1,4-PT–TIPS**.


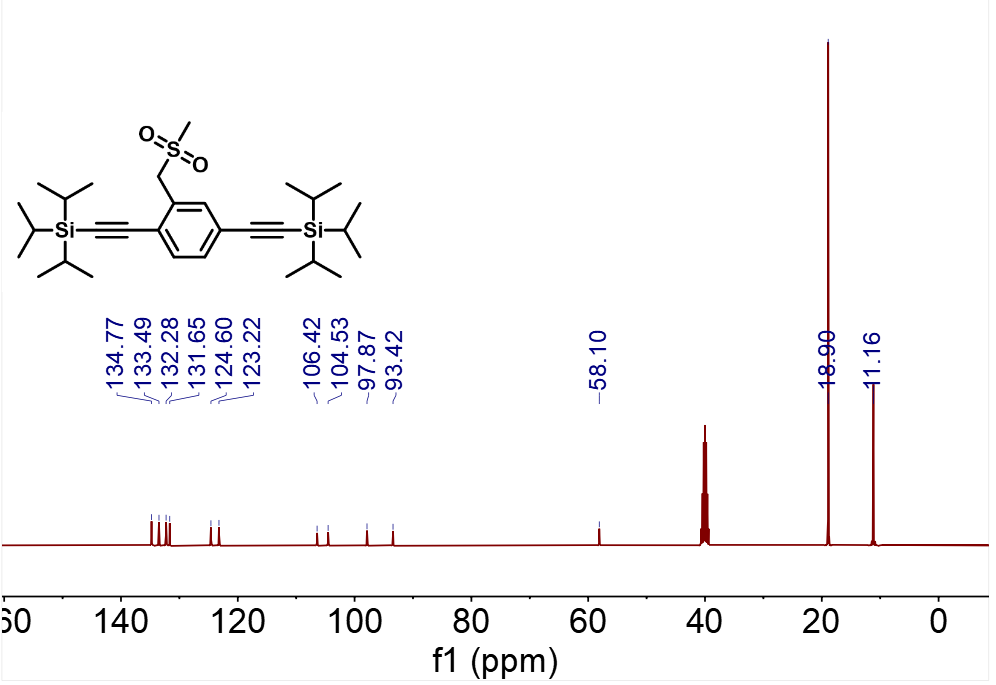


**Figure S4**. ^13^C NMR (101 MHz, DMSO-*d*_6_, 298 K) spectrum of **1,4-PT–TIPS**.

(4): *Synthesis of 1,4-diethynyl-2-((methylsulfonyl)methyl)benzene (****1,4-PT)***

((2-((methylsulfonyl)methyl)-1,4-phenylene)bis(ethyne-2,1-diyl))bis(triisopropylsilane) (2.09 g, 4 mmol) was dissolved in THF (50 mL), and then tetrabutylammonium fluoride (5.14 g, 19.6 mmol) was added dropwise at low temperature. After stirring for 30 minutes, the reaction mixture was evaporated to dryness to yield a solid product. Purification by dichloromethane column chromatography afforded 0.87g of yellow solid with a yield of 95%.

^1^H NMR (400 MHz, DMSO-*d*_6_) δ 7.61 (d, J = 2.4 Hz, 1H), 7.53 (d, J =8 Hz, 1H), 7.52（d, J = 8 Hz, 1H), 4.64 (s, 3H), 4.43 (s,1H), 2.97 (s, 3H). ^13^C NMR (101 MHz, DMSO-*d_6_*) δ 135.04, 133.50, 132.23, 131.94, 124.16, 122.66, 87.76, 83.80, 82.86, 81.24, 57.92, 40.82. *m/z*: [M]^+^ calcd for C_12_H_10_O_2_S, 218.27; found 240.87.


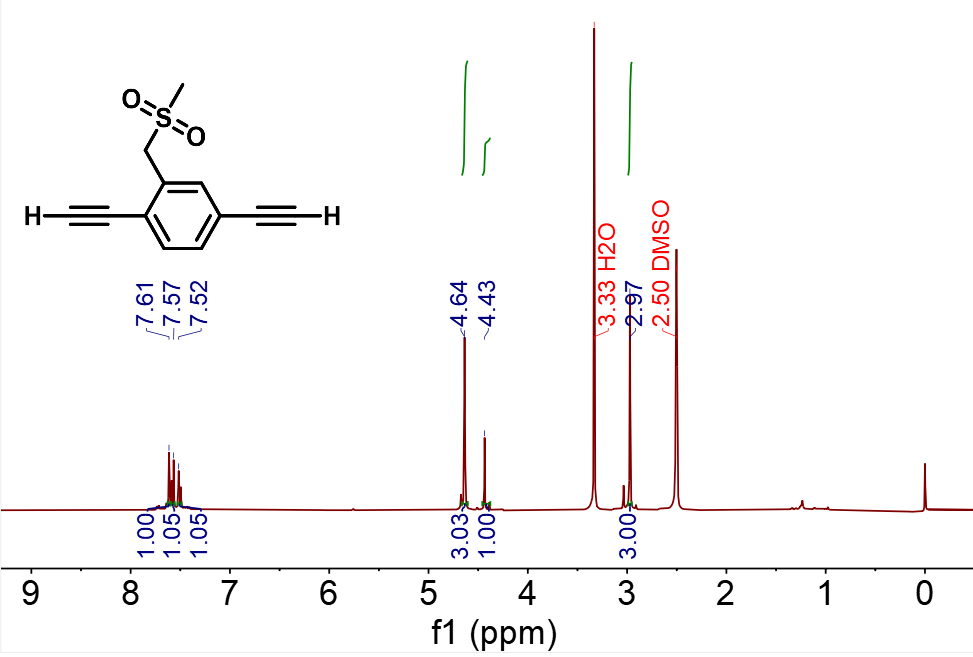


**Figure S5**. ^1^H NMR (400 MHz, DMSO-*d*_6_, 298 K) spectrum of **1,4-PT**.


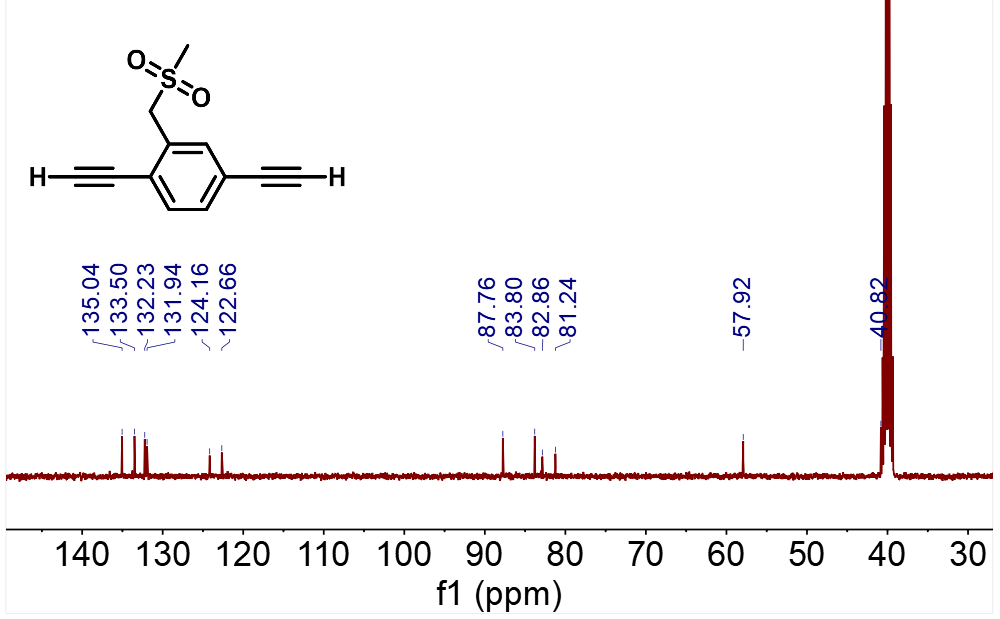


**Figure S6**. ^13^C NMR (101 MHz, DMSO-*d*_6_, 298 K) spectrum of **1,4-PT**.

# **2.2** Synthesis of 1,3-P, 1,3-PT–TIPS, 1,3-PT

**Scheme S2.** Synthetic routes of the **1,3-P**, **1,3-PT–TIPS** and **1,3-PT**.

(1): S*ynthesis of 1,3-dibromo-5-(bromomethyl) benzene*

2,6-dibromotoluene (20.06 g, 80.26 mmol), NBS (17.14 g, 96.31 mmol), AIBN (0.66 g, 4 mmol), dissolved in acetonitrile (120 ml), heated under argon protection to 85°C and refluxed for 3 hours. After cooling to room temperature, the mixture was rotary evaporated, and the residue was purified by petroleum ether column chromatography to yield 22.3 g of white crystals, with a yield of 84.5%.^[6]^

(2): *Synthesis of 1,3-dibromo-2-((methylsulfonyl) methyl) benzene (****1,3-P****)*

1,3-dibromo-2-(bromomethyl) benzene (20 g, 60.82 mmol) and sodium methanesulfinate (14.97 g, 146.64 mmol) were added to DMF (150 mL) and reacted at 70°C for 3 hours. It was cooled to room temperature, settled in saturated NaCI aqueous solution, filtered and dried to obtain 19 g white product with 95.7% yield.^[6]^

^1^H NMR (400 MHz, DMSO-*d*_6_) δ 7.77 (d, J = 8.0 Hz, 2H), 7.25 (t, J =8.0 Hz, 1H), 4.89 (s, 2H), 3.15 (s, 3H). ^13^C NMR (101 MHz, DMSO-*d*_6_) δ 138.11, 137.00, 134.40, 131.80, 66.06, 48.38. *m/z*: [M]^+^ calcd for C_8_H_8_Br_2_O_2_S, 328.02; found 351.03.


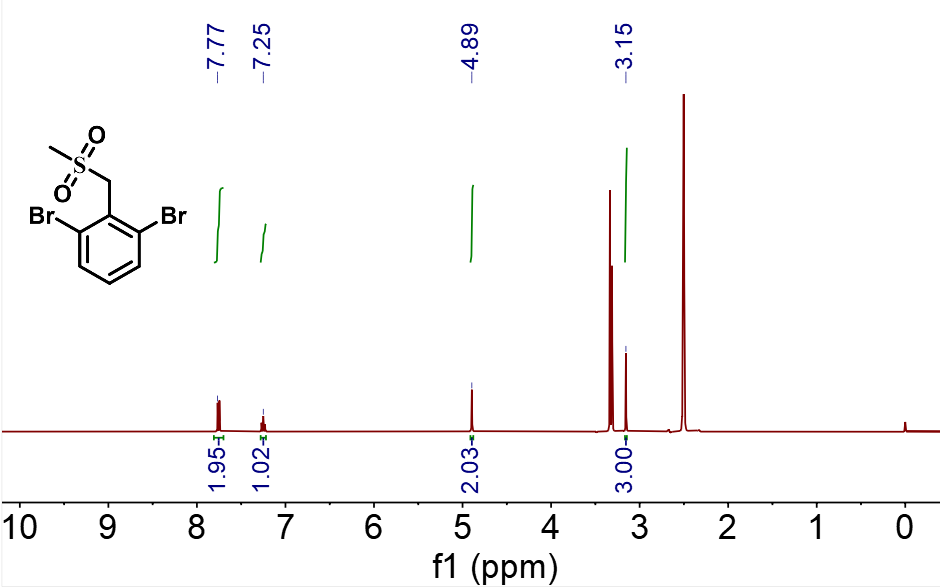


**Figure S7**. ^1^H NMR (400 MHz, DMSO-*d*_6_, 298 K) spectrum of **1,3-P**.


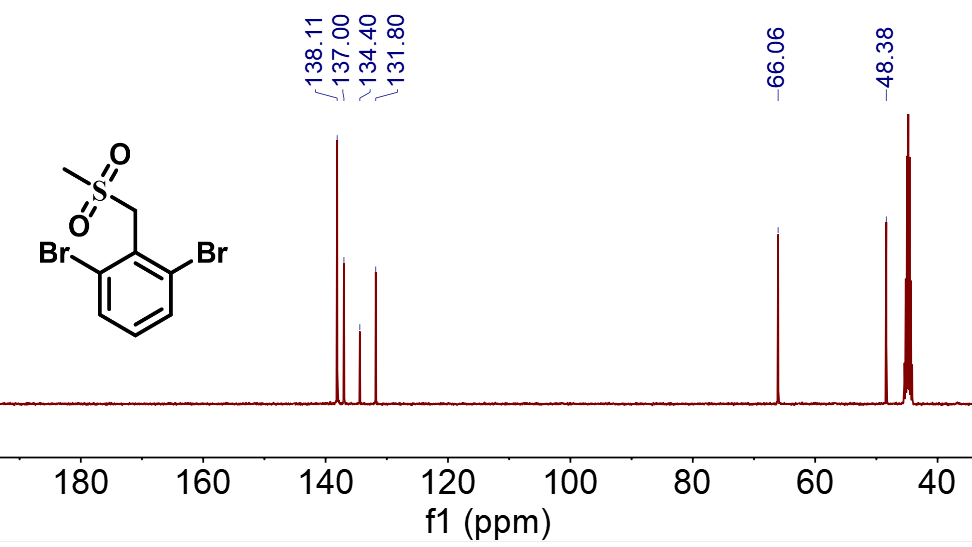


**Figure S8**. ^13^C NMR (101 MHz, DMSO-*d*_6_, 298 K) spectrum of **1,3-P**.

(3): *Synthesis of ((2-((methylsulfonyl)methyl)-1,3-phenylene)bis(ethyne-2,1-diyl))bis(triisopropylsilane) (****1,3-PT–TIPS)***

1,3-dibromo-2-((methylsulfonyl) methyl) benzene (2.116 g, 3.2mmol), triisopropyl silyl acetylene (2.882 g, 8.1 mmol), bistriphenylphosphine palladium dichloride (44 mg, 0.3 mmol), cuprous iodide (122 mg, 0.32mmol) was dissolved in piperidine (150 ml), heated to 70℃ for 24 h under the protection of argon, and then spin dried at room temperature. 2.6 g light yellow solid was obtained by dichloromethane column chromatography with a yield of 92.8 %.

^1^H NMR (400 MHz, DMSO-*d*_6_) δ 7.62 (d, J = 8.0 Hz, 1H), 7.43 (t, J =8.0 Hz, 1H), 4.76 (s, 2H), 2.96 (s, 3H). 1.11 (s, 42H).^13^C NMR (101 MHz, DMSO-*d*_6_) δ 134.07, 132.45, 129.64, 125.22, 104.76, 96.46, 58.91, 41.85, 18.92, 11.18. *m/z*: [M]^+^ calcd for C_30_H_50_O_2_SSi_2_, 530.96; found 553.30.


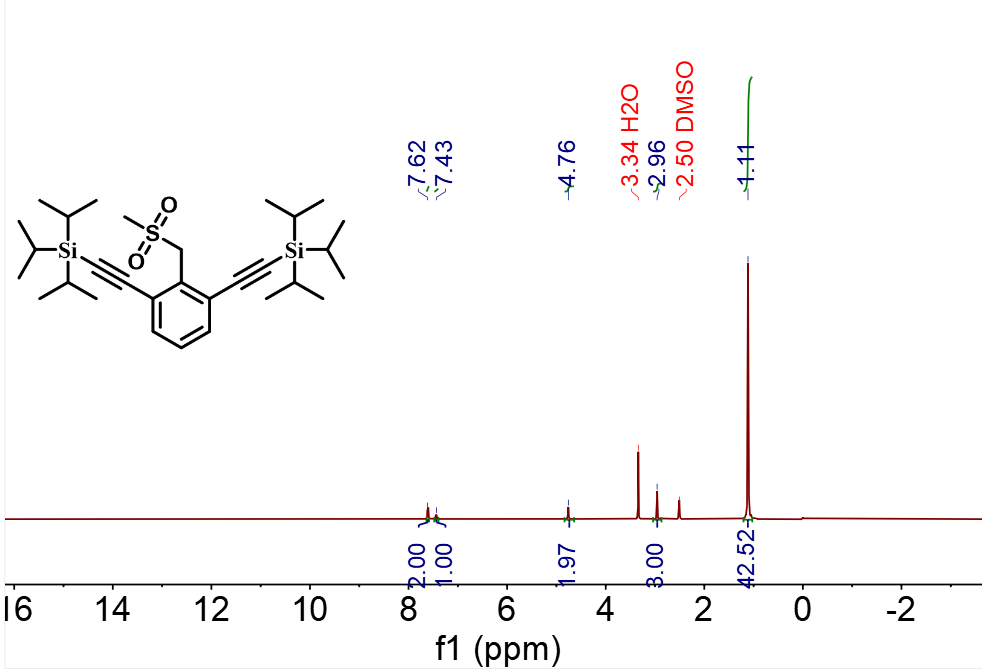


**Figure S9**. ^1^H NMR (400 MHz, DMSO-*d*_6_, 298 K) spectrum of **1,3-PT-TIPS**.


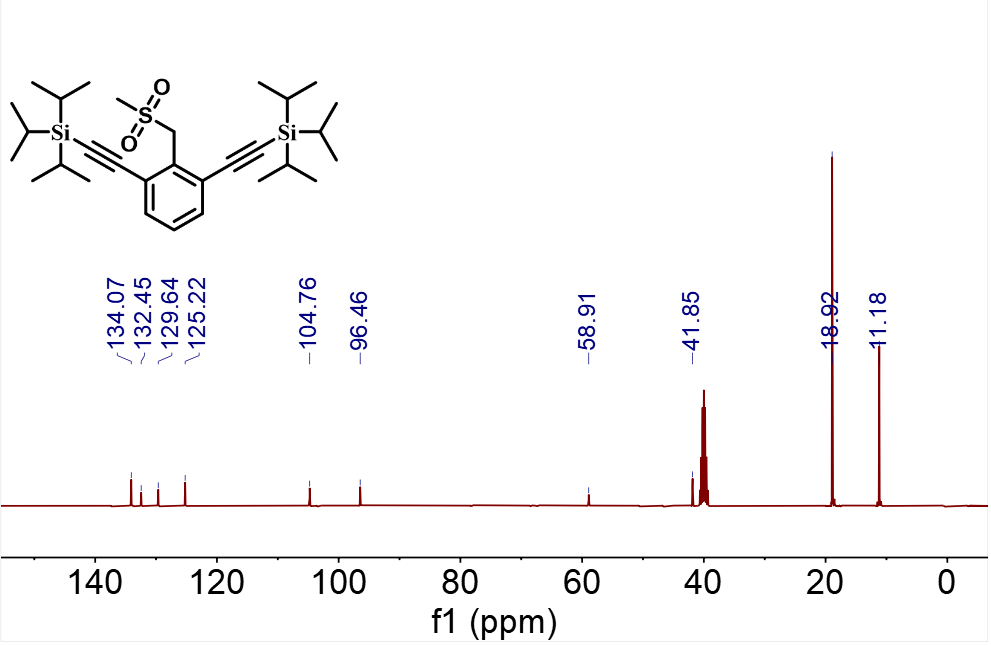


**Figure S10**. ^13^C NMR (101 MHz, DMSO-*d*_6_, 298 K) spectrum of **1,3-PT-TIPS**.

(4): *Synthesis of 1,3-diethynyl-2-((methylsulfonyl)methyl)benzene (****1,3-PT)***

((2-((methylsulfonyl)methyl)-1,3-phenylene)bis(ethyne-2,1-diyl))bis(triisopropylsilane) (2.09 g, 4 mmol) was dissolved in THF (50 mL), and then tetrabutylammonium fluoride (5.14 g, 19.6 mmol) was added dropwise at low temperature. After stirring for 30 minutes, the reaction mixture was evaporated to dryness to yield a solid product. Purification by dichloromethane column chromatography afforded 0.88g of yellow solid with a yield of 95%.

^1^H NMR (400 MHz, DMSO-*d*_6_) δ 7.63 (d, J = 8.0 Hz, 1H), 7.43 (t, J =8 Hz, 1H), 4.76 (s, 2H), 4.56 (s,2H), 3.03 (s, 3H). ^13^C NMR (101 MHz, DMSO-*d*_6_) δ 133.85, 133.60, 129.51, 124.63, 86.63, 81.61, 58.53, 42.81. *m/z*: [M]^+^ calcd for C_12_H_10_O_2_S, 218.27; found 241.05.


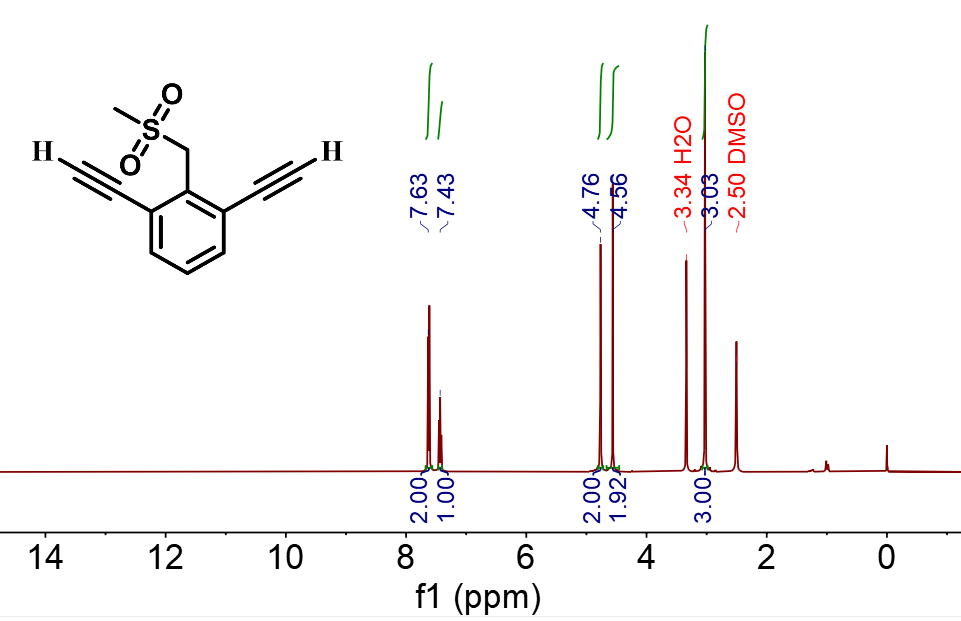


**Figure S11**. ^1^H NMR (400 MHz, DMSO-*d*_6_, 298 K) spectrum of **1,3-PT**.


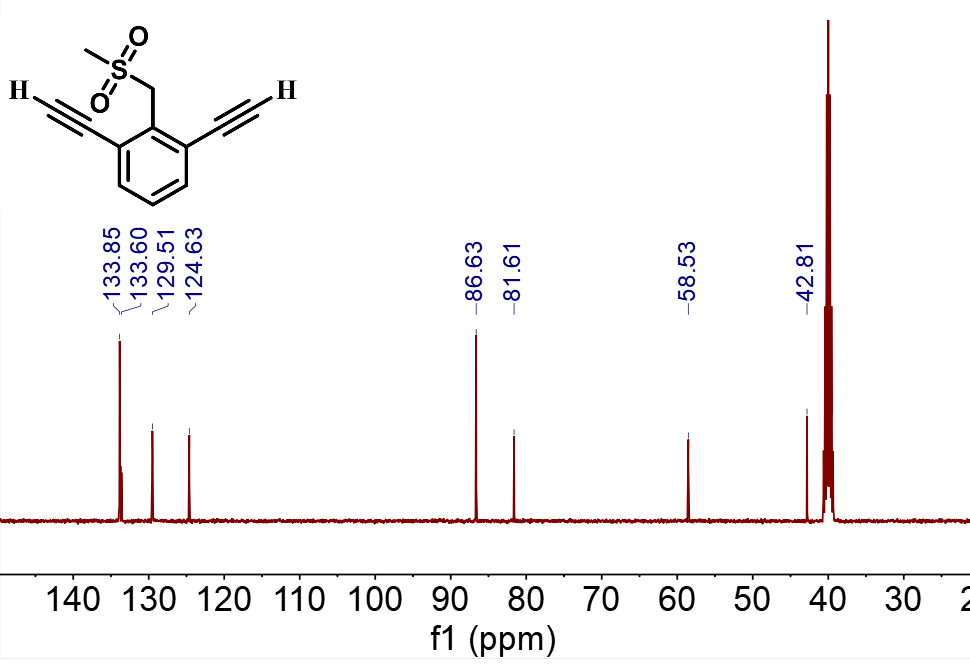


**Figure S12**. ^13^C NMR (101 MHz, DMSO-*d*_6_, 298 K) spectrum of **1,3-PT**.

## 2.3 Synthesis of compound P-PTAQ

**Scheme S3.** Synthetic route of the **P-PTAQ**.

## 1,4-PT (64 mg) and 2,6-dibromoanthraquinone (50 mg) were dissolved in 10 mL of dimethylformamide (DMF) under an argon atmosphere. (PPh_3_)_2_PdCl_2_ (13 mg), CuI (3.71 mg), and triethylamine (8 mL) were added to the solution. The reaction mixture was stirred at 80°C for 48 hours. After cooling, the catalyst was washed with DMF and then centrifuged three times. Then it was washed ultrasonically with ethanol for three times. The catalyst was filtered and then dried at 60°C, with a synthetic yield of 87%.

## 2.4 Synthesis of compound O-PTAQ

**Scheme S4.** Synthetic route of the **O-PTAQ**.

1,3-PT (64 mg) and 2,6-dibromoanthraquinone (50 mg) were dissolved in 10 mL of dimethylformamide (DMF) under an argon atmosphere. Then, (PPh_3_)_2_PdCl_2_ (13 mg), CuI (3.71 mg), and triethylamine (8 mL) were added to the solution. The reaction mixture was stirred at 80 °C for 48 hours. After cooling, the catalyst was washed with DMF and centrifuged three times, followed by ultrasonic washing with ethanol for three times. Subsequently, the catalyst was filtered and dried at 60 °C.The yield was 83%.


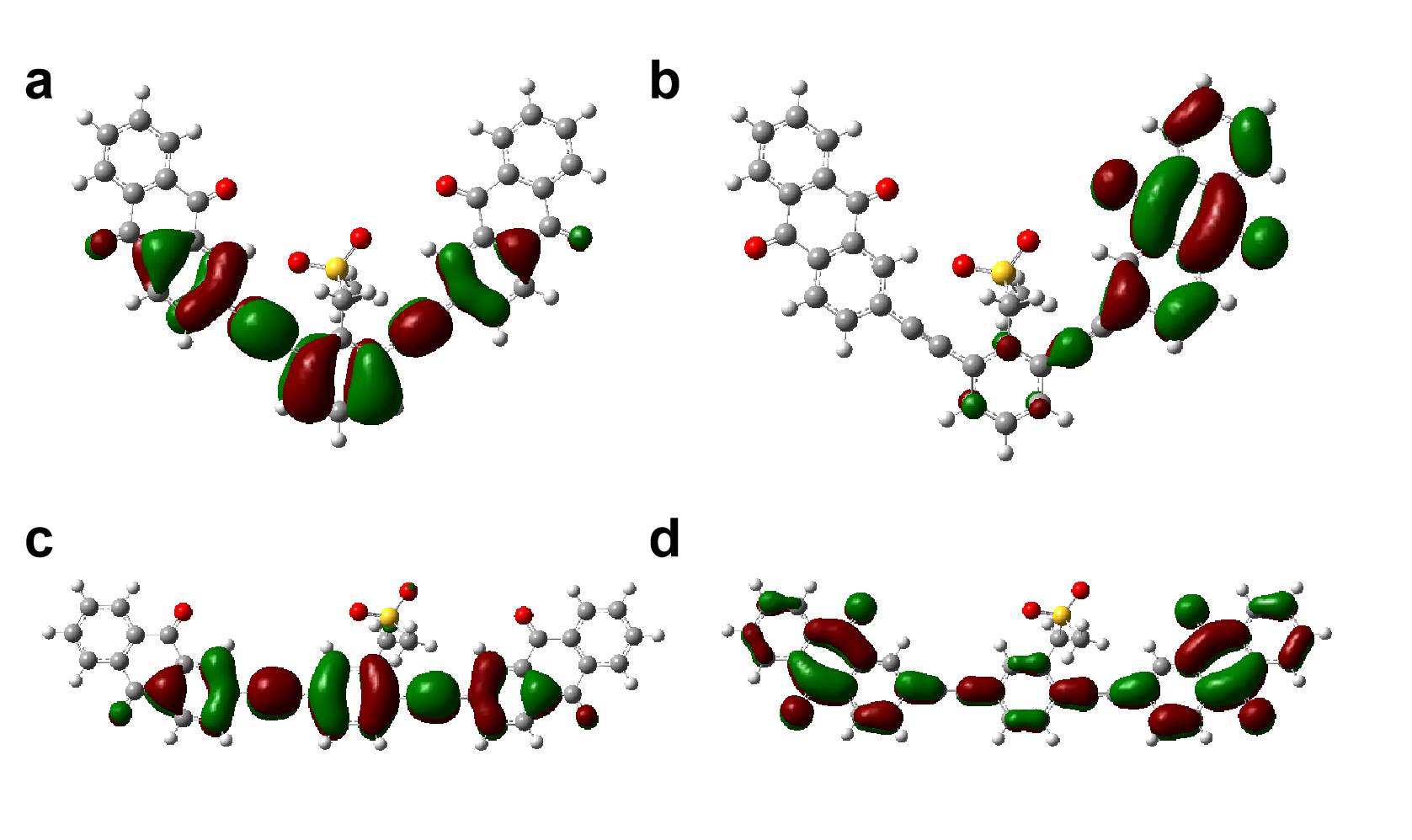


**Figure S13.** The highest occupied molecular orbital (HOMO) diagrams of (a) O-PTAQ and (c) P-PTAQ. The lowest unoccupied molecular orbital (LUMO) diagrams of (b) O-PTAQ and (d) P-PTAQ.


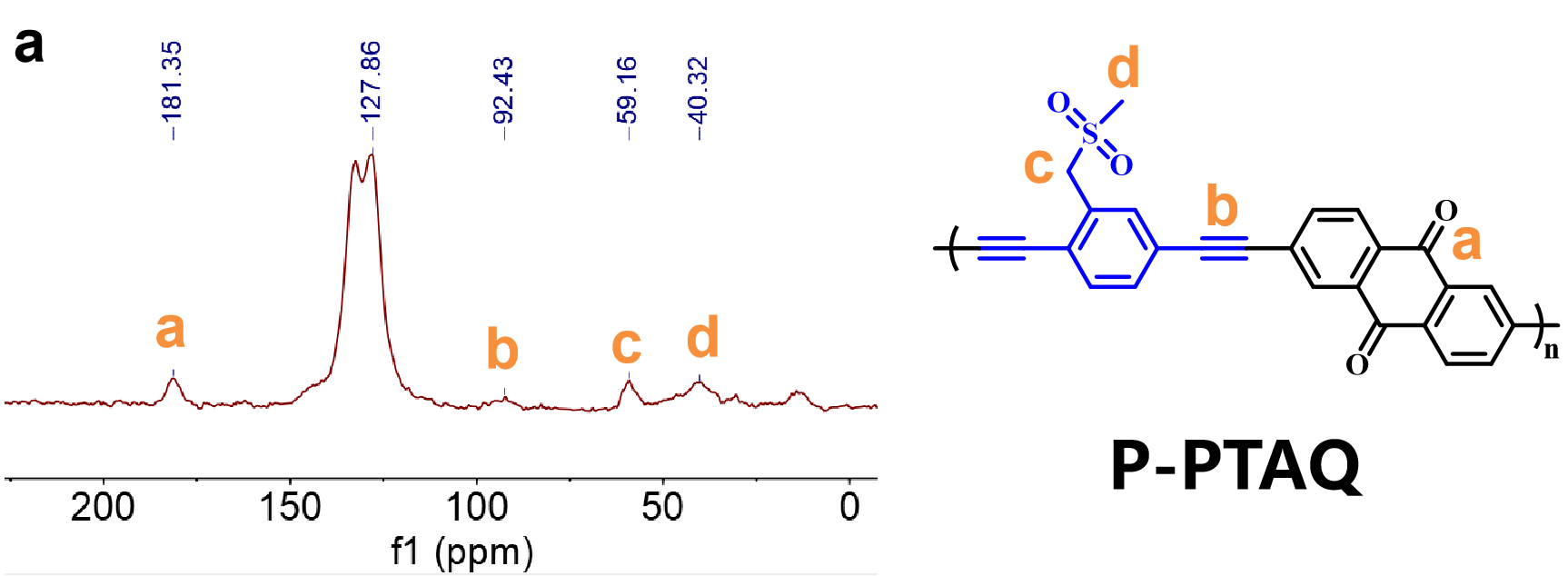


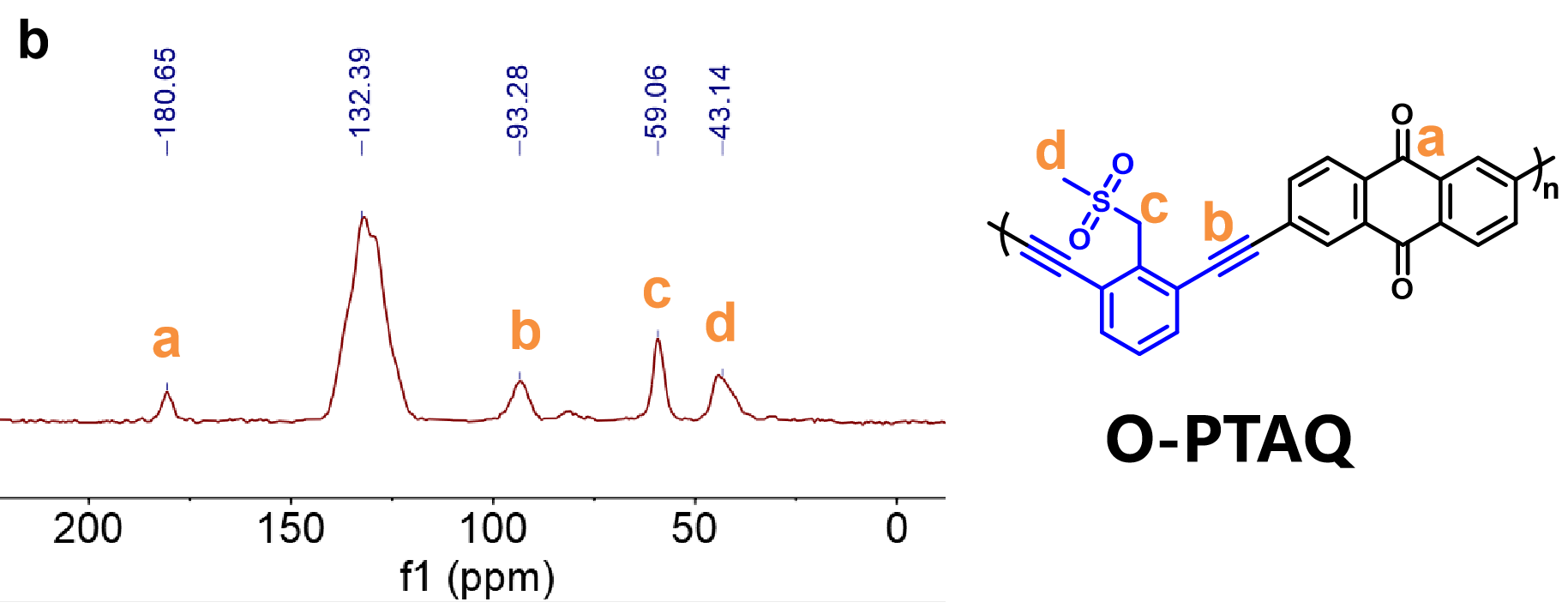


**Figure S14.** Solid-state ^13^C NMR spectra of (a) P-PTAQ and (b) O-PTAQ.

**Figure S15.** XPS spectra of P-PTAQ (a), O-PTAQ (b), for C 1s and P-PTAQ (c), O-PTAQ (d), for S 2p.

**Figure S16.** FT-IR spectra of CPs.

**Figure S17.** Raman spectra of O-PTAQ and P-PTAQ.

**Figure S18.** The PXRD characterization for CPs.

**Figure S19.** SEM images of O-PTAQ (a) and P-PTAQ (b).

**Figure S20.** TEM image of P-PTAQ and its energy dispersive spectroscopy (EDS) mapping images.

**Figure S21.** TEM image of O-PTAQ and its energy dispersive spectroscopy (EDS) mapping images.

**Figure S22.** N_2_ adsorption-desorption isotherms of O-PTAQ and P-PTAQ.

**Figure S23.** TG curves of O-PTAQ and P-PTAQ under N_2_ atmosphere.

**Figure S24.** Mott-Schottky plots of (a) P-PTAQ, (b) O-PTAQ for characterizing conduction band (CB).

**Figure S25.** XPS valance band spectra of (a) P-PTAQ, (b) O-PTAQ.

**Figure S26.** The work function testing with KPFM for (a) Au, (b) P-PTAQ and (c) O-PTAQ. The determination of the work function (φ) involves calibration with a gold standard sample. The calculation formula for the work function (φ) is: φ = 5.2 - (V_R_ - V_Au_), where V_R_ is the measured potential of the material and V_Au_ is the potential of the gold standard sample.

**Figure S27.** The apparent quantum yields for H_2_O_2_ generation of O-PTAQ and P-PTAQ at the specified wavelength.

**Figure S28.** Comparison between the O-PTAQ and P-PTAQ after 5 cycles of photoreaction: (a, b) PXRD image and (c, d) FT-IR spectra.

**Figure S29.** Photosynthetic H_2_O_2_ production by CPs in pure water, lake water, and the Pearl River over one hour.

**Figure S30.** Photocatalytic H_2_O_2_ production of P-PTAQ and O-PTAQ in Ar and Air.


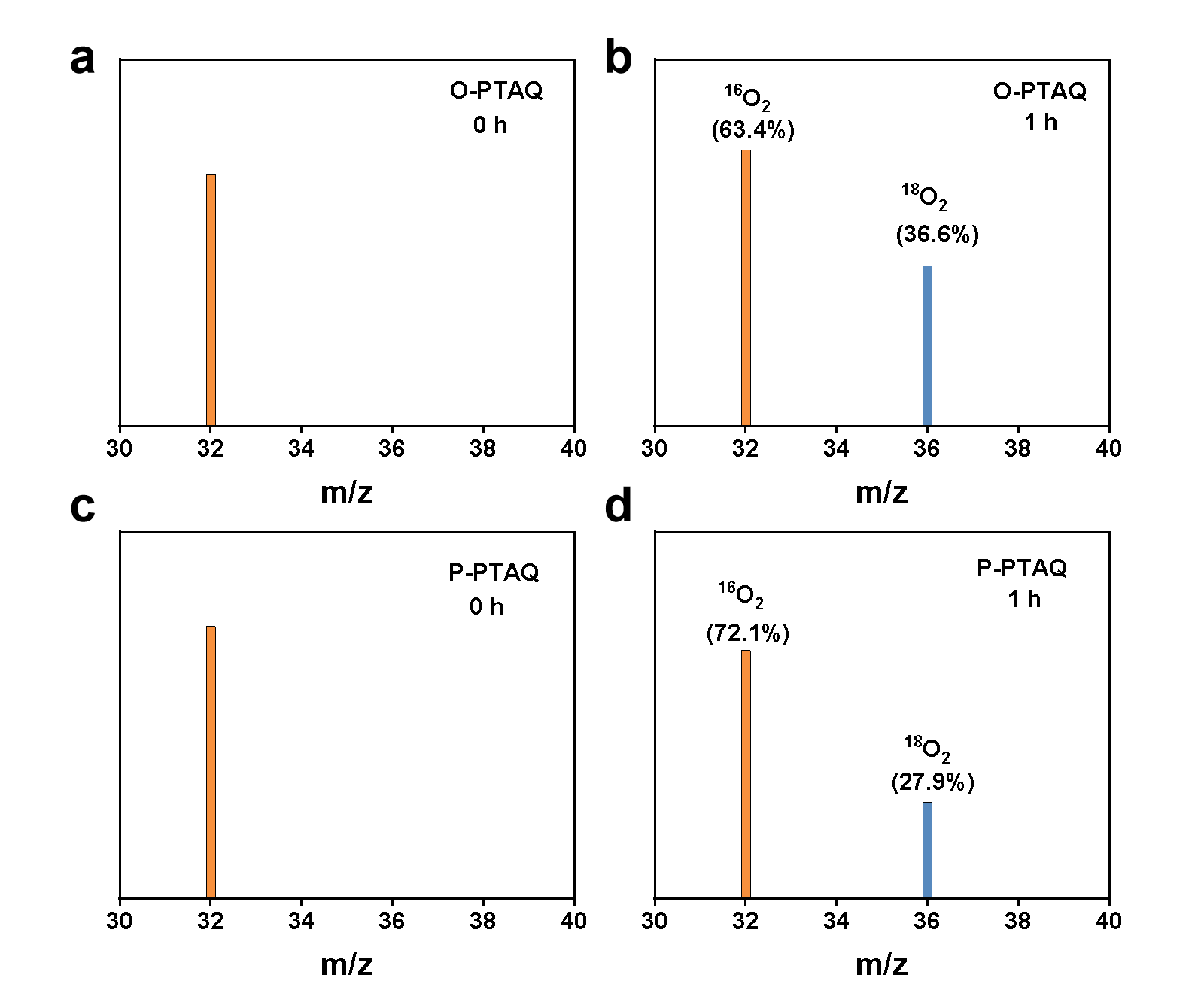


**Figure S31.** Isotope experiments and tests of CPs. (1 mg of the catalyst was added to H_2_^18^O in an air atmosphere. The generated H_2_O_2_ was decomposed using manganese dioxide.)


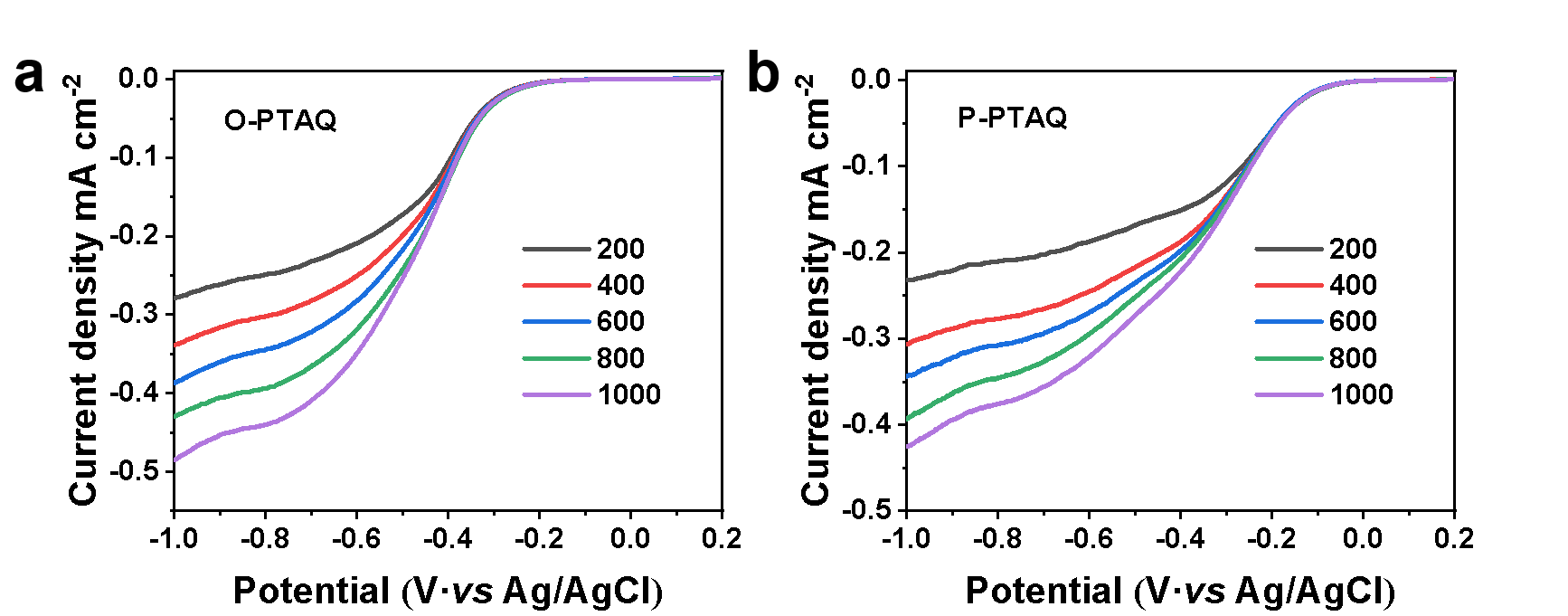


**Figure S32.** The LSV curves were measured on RDE at different rotating of (a) O-PTAQ and (b) P-PTAQ.

**Figure S33.** Koutecky–Levich plots obtained by RDE measurements.


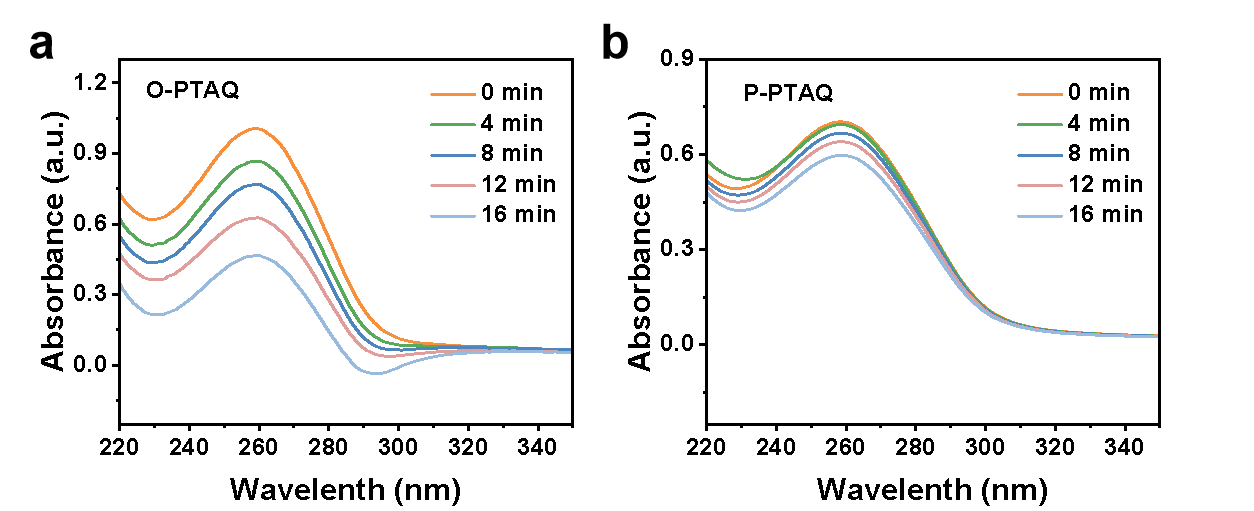


**Figure S34.** The absorption changes of NBT with (a) O-PTAQ and (b) P-PTAQ.


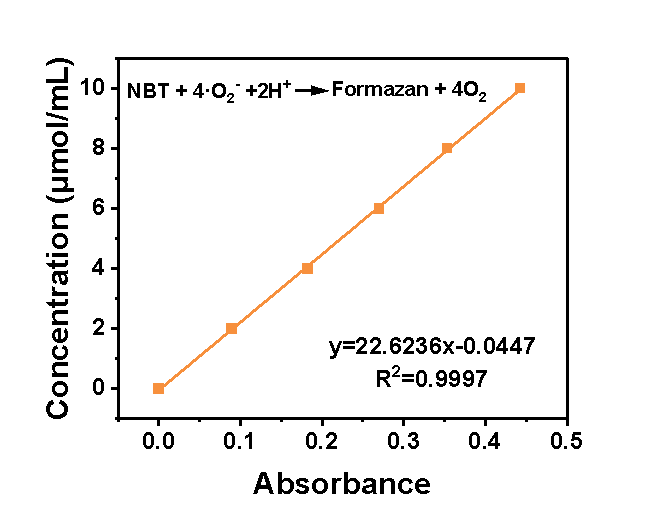


**Figure S35.** Standard curve of •O_2_^-^.

**Figure S36.** O_2_ adsorption sites and adsorption energy for O-PTAQ.


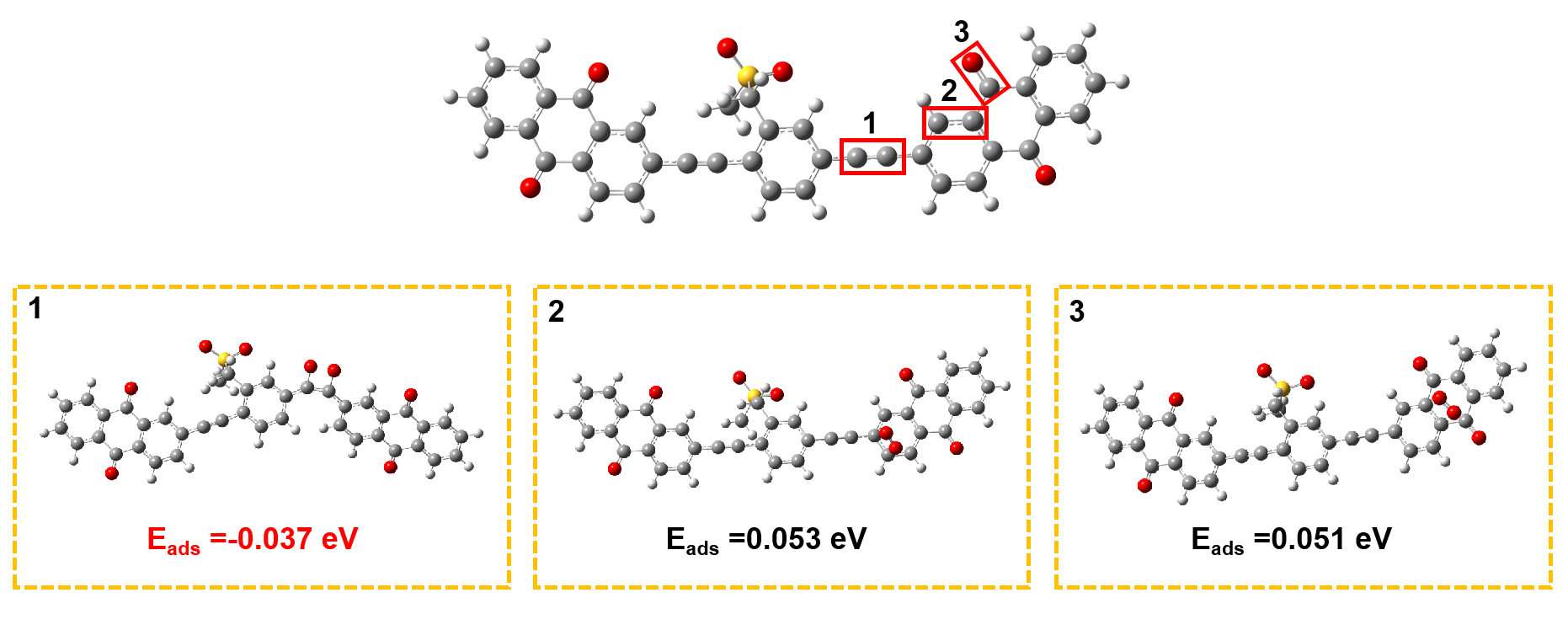


**Figure S37.** O_2_ adsorption sites and adsorption energy for P-PTAQ.


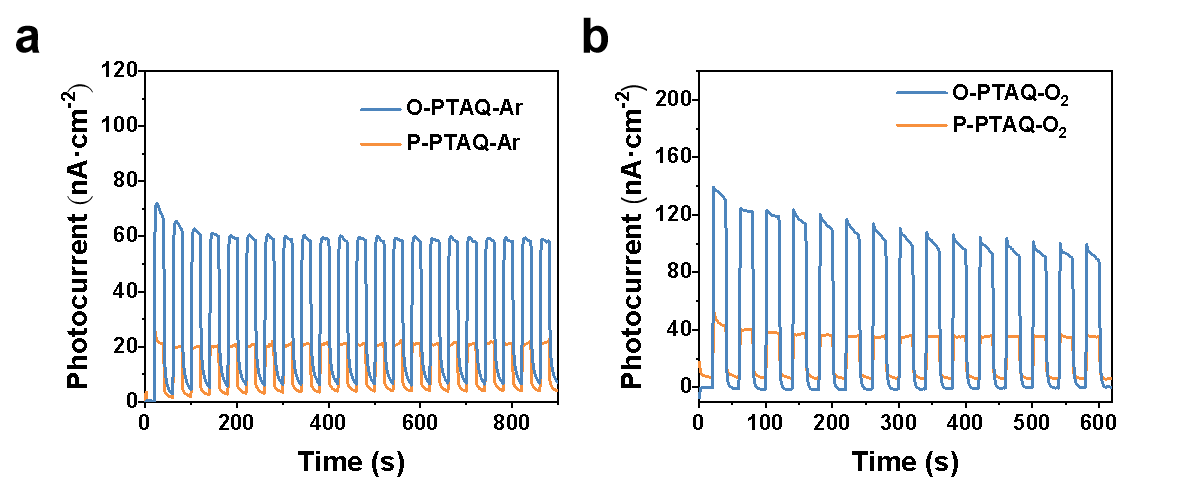


**Figure S38.** Comparison of transient photocurrent responses for O-PTAQ and P-PTAQ in (a) Ar and (b) oxygen.


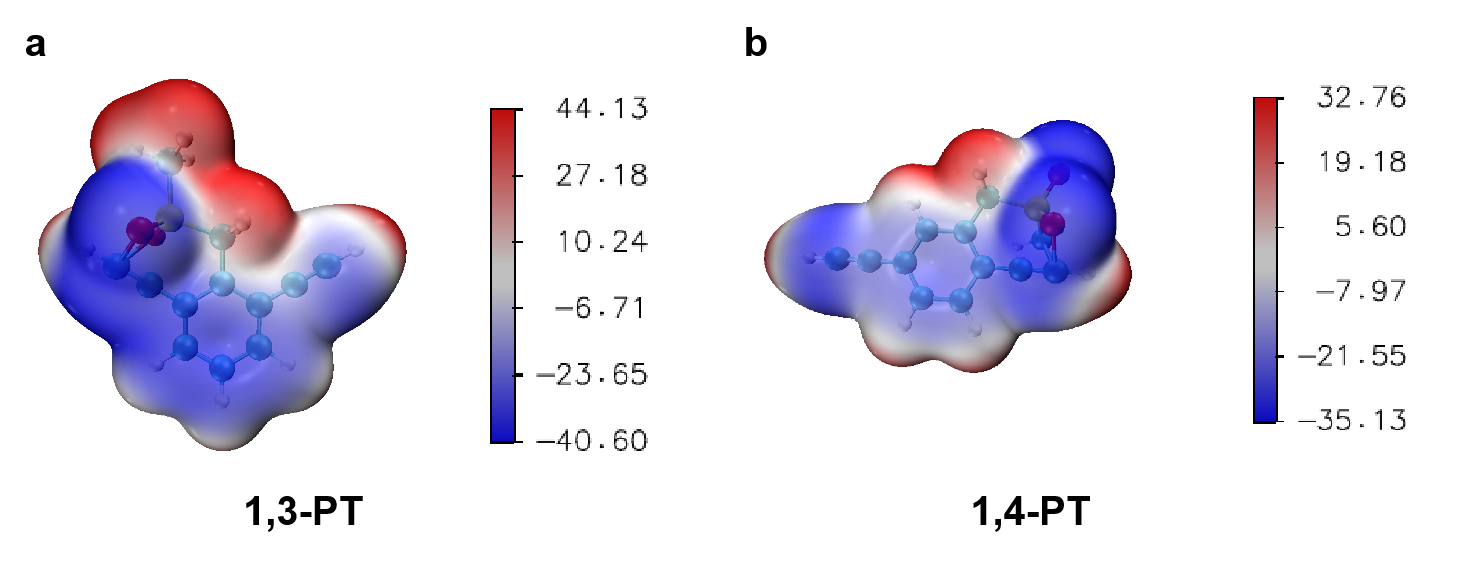


**Figure S39.** Electrostatic potential (ESP) of 1,3-PT and 1,4-PT.


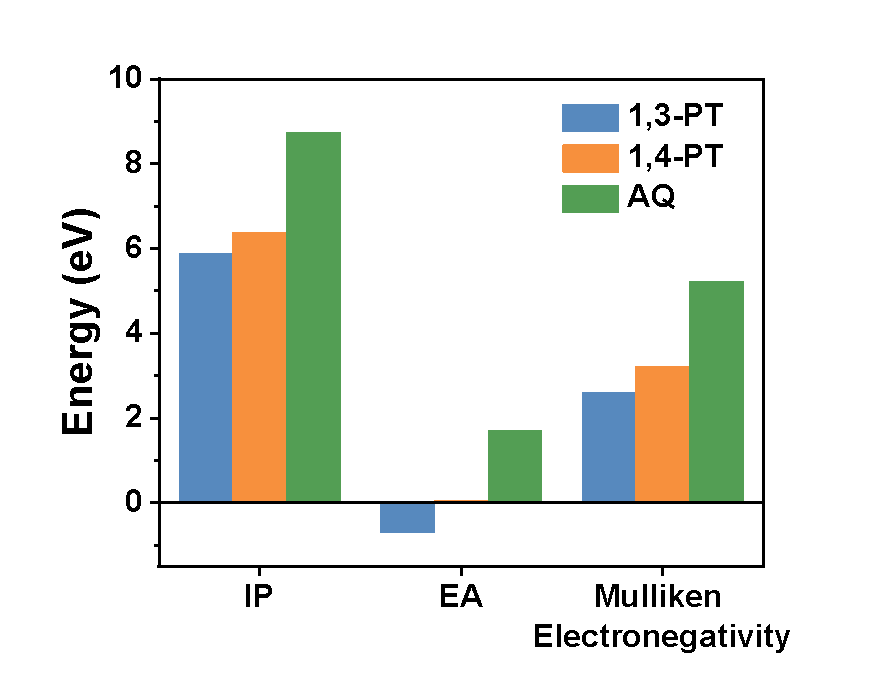


**Figure S40.** Calculation of the ionization potential (IP), electron affinity (EA), and Mulliken electronegativity of 1,3-PT, 1,4-PT and AQ monomers. Mulliken electronegativity = (IP+EA)/2.

**Figure S41.** Time slices of the TA spectra for P-PTAQ in AgNO_3_ (a) and EDTA-2Na (b), and for O-PTAQ in AgNO_3_ (c) and EDTA-2Na (d). The concentrations of EDTA-2Na and AgNO_3_ utilized were both 10 mM.

**Figure S42.** Electrochemical impedance spectra of O-PTAQ and P-PTAQ.

# Supplementary Tables

**Table S1.** Brunauer-Emmett-Teller (BET) surface areas of the CPs

|  | **O-PTAQ** | **P-PTAQ** |
| --- | --- | --- |
| BET Surface (m^2^·g^-1^) | 205.26 | 87.15 |

**Table S2.** The comparison of the hydrogen peroxide yield between O-PTAQ and other reported organic photocatalysts.

| **Catalyst** | | **Water**  **body** | **Atmosphere** | **Light**  **Intensity/mW·cm^-2^** | **H_2_O_2_ yield**  **/μmol·g^-1^·h^-1^** | **AQY(%) at 420nm** | **Ref.** |
| --- | --- | --- | --- | --- | --- | --- | --- |
| TPE-AQ | pure water | | Air | 100  (λ > 400 nm) | 909 | / | ^[7]^ |
| KDBT | pure water | | Air | 100  (λ > 400 nm) | 2368 | / | ^[8]^ |
|  | pure water | | Air | ambient  conditions | 2676 | / |  |
| TPT-alkynyl-AQ | river water | | Air | ambient  conditions | 2464 | 18 (425nm) | ^[9]^ |
|  | seawater | | Air | ambient  conditions | 2746 | / |  |
| TTP | pure water | | Air | 300 | 3132 | 7.61 | ^[10]^ |
| SA-SADF-H^+^ | pure water | | O_2_ | 100  (λ > 420 nm) | 4666.7 | 25.9 | ^[11]^ |
| COF-N32 | pure water | | Air | 100  (λ > 420 nm) | 605 | 5.5 | ^[12]^ |
| FS-COFs | pure water | | O_2_ | LED irradiation  (λ > 400 nm) | 3904.2 | 6.21 | ^[13]^ |
| TZ-COF | pure water | | Air | (λ > 420 nm) | 268 | / | ^[14]^ |
| TaptBtt | pure water | | Air | 300  (λ > 420 nm) | 1407 | 4.4 | ^[15]^ |
| TF50 -COF | ethanol | | Air | (λ > 400 nm) | 1739 | 5.1 (400nm) | ^[16]^ |
| o-COF-TpPzda | pure water | | O_2_ | 100 | 4396 | / | ^[17]^ |
| Bpt-CTF | pure water | | O_2_ | 300 | 3268.1 | 8.6 (400nm) | ^[18]^ |
| O-PTAQ | pure water | | Air | 100  (λ > 400 nm) | 4989 | 26.6 | This  work |

**Table S3.** Solar-to-chemical conversion (SCC) efficiencies for various photocatalytic reactions in recent literature.

| Photocatalysts | Conditions | | | SCC efficiency | Ref. |
| --- | --- | --- | --- | --- | --- |
|  | Atmosphere | Water body | |  |  |
| TapbBtt | O_2_ | Pure water | | 0.297% | ^[15]^ |
| PyIm-COF | O_2_ | | Pure water | 0.28% | ^[19]^ |
| BTDB-CN_0.2_ | Air | | Pure water | 0.019 % | ^[20]^ |
| CTF-FL | Air | | Pure water | 0.91 | ^[21]^ |
| SO_3_H-COF | Air | | Pure water | 0.4% | ^[22]^ |
| CHF-DPDA | O_2_ | | Pure water | 0.78% | ^[23]^ |
| HEP-TAPT-COF | O_2_ | | Pure water | 0.65% | ^[24]^ |
| TpDz | Air | | Pure water | 0.62% | ^[25]^ |
| RF-DHAQ-2 | O_2_ | | Pure water | 1.2% | ^[26]^ |
| SA-TCPP | O_2_ | | Pure water | 1.2% | ^[27]^ |
| TD-COF | Air | | Seawater | 0.15% | ^[28]^ |
| TTF-BT-COF | O_2_ | | Pure water | 0.49% | ^[29]^ |
| *β*-TT-TDAN | O_2_ | | Pure water | 1.35% | ^[30]^ |
| PIC-BPY | Air | | Pure water | 0.22% | ^[31]^ |
| O-PTAQ | Air | | Pure water | 1.64% | This work |

**Table S4.** The surface analysis of CPs.

|  | **O-PTAQ** | **P-PTAQ** |
| --- | --- | --- |
| Internal charge separation (Pi)/kcal·mol^-1^ | 15.11 | 13.12 |
| Molecular polarity index (MPl)/kcal·mol^-1^ | 14.95 | 13.21 |

**Table S5.** Calculation of the ionization potential (IP), electron affinity (EA), and Mulliken electronegativity of 1,3-PT, 1,4-PT and AQ monomers. Mulliken electronegativity = (IP+EA)/2.

|  | **IP (eV)** | **EA (eV)** | **Mulliken Electronegativity (eV)** |
| --- | --- | --- | --- |
| 1,3-PT | 5.8882 | -0.7039 | 2.5922 |
| 1,4-PT | 6.3886 | 0.0488 | 3.2187 |
| AQ | 8.7499 | 1.7083 | 5.2291 |

[1] Grimme S, Antony J, Ehrlich S, et al. A consistent and accurateab initioparametrization of density functional dispersion correction (DFT-D) for the 94 elements H-Pu. J.Chem.Phys. 2010, 132, 154104.

[2] Krishnan R, Binkley J S, Seeger R, et al. Self-consistent molecular orbital methods. A basis set for correlated wave functions. J.Chem.Phys. 1980, 72, 650-654.

[3] P. J. Stephens F J D Ab Initio Calculation of Vibrational Absorption and Circular Dichroism Spectra Using Density Functional Force Fields. Phys. Chem. 1994, 98, 11623-11627.

[4] Machura B, Gryca I, Małecki J G, et al. p-Tolylimido rhenium(v) complexes-synthesis, X-ray studies, spectroscopic characterization, DFT calculations and catalytic activity. Dalton Trans. 2014, 43, 2596-2610.

[5] Liu Z, Lu T ,Chen Q An sp-hybridized all-carboatomic ring, cyclo[18]carbon: Electronic structure, electronic spectrum, and optical nonlinearity. Carb. 2020, 165, 461-467.

[6] Zheng W, Yang T, Qu L, et al. Temperature resistant amorphous polyimides with high intrinsic permittivity for electronic applications. Chem. Eng. J. 2022, 436, 135060.

[7] Ye Y-X, Pan J, Shen Y, et al. A solar-to-chemical conversion efficiency up to 0.26% achieved in ambient conditions. Proc. Natl. Acad. Sci. 2021, 118,

[8] Cheng C, Yu J, Xu D, et al. In-situ formatting donor-acceptor polymer with giant dipole moment and ultrafast exciton separation. Nat. Commun. 2024, 15, 1313.

[9] Yan H，Shen M, Shen Y, et al. Spontaneous exciton dissociation in organic photocatalyst under ambient conditions for highly efficient synthesis of hydrogen peroxide. Proc. Natl. Acad. Sci. 2022, 119, e2202913119.

[10] Chu C, Chen Z, Yao D, et al. Large-Scale Continuous and In Situ Photosynthesis of Hydrogen Peroxide by Sulfur-Functionalized Polymer Catalyst for Water Treatment. Angew. Chem. Int. Ed. 2024, 63, e202317214.

[11] Li W, Han B, Liu Y, et al. Unsymmetric Protonation Driven Highly Efficient H_2_O_2_ Photosynthesis in Supramolecular Photocatalysts via One-Step Two-Electron Oxygen Reduction. Angew. Chem. Int. Ed. 2024, e202421356.

[12] Liu F, Zhou P, Hou Y, et al. Covalent organic frameworks for direct photosynthesis of hydrogen peroxide from water, air and sunlight. Nat. Commun. 2023, 14, 4344

[13] Luo Y, Zhang B, Liu C, et al. Sulfone-Modified Covalent Organic Frameworks Enabling Efficient Photocatalytic Hydrogen Peroxide Generation via One-Step Two-Electron O_2_ Reduction. Angew. Chem. Int. Ed. 2023, 62, e202305355.

[14] Mou Y, Wu X, Qin C, et al. Linkage Microenvironment of Azoles-Related Covalent Organic Frameworks Precisely Regulates Photocatalytic Generation of Hydrogen Peroxide. Angew. Chem. Int. Ed. 2023, 62, e202309480.

[15] Qin C, Wu X, Tang L, et al. Dual donor-acceptor covalent organic frameworks for hydrogen peroxide photosynthesis. Nat. Commun. 2023, 14, 5238

[16] Wang H, Yang C, Chen F, et al. A Crystalline Partially Fluorinated Triazine Covalent Organic Framework for Efficient Photosynthesis of Hydrogen Peroxide. Angew. Chem. Int. Ed. 2022, 61, e202202328.

[17] Yang T, Zhang D, Kong A, et al. Robust Covalent Organic Framework Photocatalysts for H2O2 Production: Linkage Position Matters. Angew. Chem. Int. Ed. 2024, 63, e202404077.

[18] Cheng H, Lv H, Cheng J, et al. Rational Design of Covalent Heptazine Frameworks with Spatially Separated Redox Centers for High-Efficiency Photocatalytic Hydrogen Peroxide Production. Adv. Mater. 2021, 34, 2107480.

[19] W. Wu, Z. Li, S. Liu, et al. Pyridine-Based Covalent Organic Frameworks with Pyridyl-Imine Structures for Boosting Photocatalytic H_2_O_2_ Production via One-Step 2e^-^ Oxygen Reduction. *Angew. Chem. Int. Ed.* 2024, *63,* e202404563.

[20] J. Cheng, W. Wang, J. Zhang, S. et al. Molecularly Tunable Heterostructured Co-Polymers Containing Electron-Deficient and -Rich Moieties for Visible-Light and Sacrificial-Agent-Free H_2_O_2_ Photosynthesis. *Angew. Chem. Int. Ed.* 2024, *63,* e202406310.

[21] Zhang L, Wang C, Jiang Q, et al. Structurally Locked High-Crystalline Covalent Triazine Frameworks Enable Remarkable Overall Photosynthesis of Hydrogen Peroxide. J. Am. Chem. Soc. 2024, 146, 43, 29943-29954.

[22] Li L, Lv X, et al. Custom-Design of Strong Electron/Proton Extractor on COFs for Efficient Photocatalytic H2O2 Production. Angew. Chem. Int. Ed. 2024, 63, e202320218.

[23] H. Cheng, H. Lv, J. Cheng, L. et al. Rational Design of Covalent Heptazine Frameworks with Spatially Separated Redox Centers for High-Efficiency Photocatalytic Hydrogen Peroxide Production. *Adv. Mater.* 2021, *34,* 2107480.

[24] D. Chen, W. Chen, Y. Wu, L. et al. Covalent Organic Frameworks Containing Dual O_2_ Reduction Centers for Overall Photosynthetic Hydrogen Peroxide Production. *Angew. Chem. Int. Ed.* 2023, *62,* e202217479.

[25] Liao Q, Sun Q, Xu H, et al. Regulating Relative Nitrogen Locations of Diazine Functionalized Covalent Organic Frameworks for Overall H_2_O_2_ Photosynthesis. Angew. Chem. Int. Ed. 2023, 62, e202310556.

[26] Zhao C, Wang X, Yin Y, et al. Molecular Level Modulation of Anthraquinone-containing Resorcinol-formaldehyde Resin Photocatalysts for H_2_O_2_ Production with Exceeding 1.2 % Efficiency. Angew. Chem. Int. Ed. 2023, 62, e202218318.

[27] Zhang Y, Pan C, Bian G, et al. H_2_O_2_ generation from O_2_ and H_2_O on a near-infrared absorbing porphyrin supramolecular photocatalyst. Nat. Energy 2023, 8, 361-371.

[28] Yue J, Song L, Fan Y, et al. Thiophene-Containing Covalent Organic Frameworks for Overall Photocatalytic H_2_O_2_ Synthesis in Water and Seawater.Angew. Chem. Int. Ed. 2023, 62, e202309624.

[29] Chang J, Li Q, Shi W, et al. Oxidation-Reduction Molecular Junction Covalent Organic Frameworks for Full Reaction Photosynthesis of H_2_O_2_. Angew. Chem. Int. Ed. 2023, 62, e202218868.

[30] Zhang W, Sun M, Cheng J, Regulating Electron Distribution in Regioisomeric Covalent Organic Frameworks for Efficient Solar-Driven Hydrogen Peroxide Production. Adv. Mater. 2025, 2500913.

[31] Kim T, Lee D-Y, Choi E, et al. Simultaneous photocatalytic hydrogen peroxide production and pollutant degradation via bipyridine-based polyimide covalent organic framework. Appl. Catal. B: Environ. Energy 2024, 357, 124264.
